# A User's Guide to a Data Base of the Diversity of *Pseudomonas syringae* and Its Application to Classifying Strains in This Phylogenetic Complex

**DOI:** 10.1371/journal.pone.0105547

**Published:** 2014-09-03

**Authors:** Odile Berge, Caroline L. Monteil, Claudia Bartoli, Charlotte Chandeysson, Caroline Guilbaud, David C. Sands, Cindy E. Morris

**Affiliations:** 1 INRA, UR0407 Pathologie Végétale, Montfavet, France; 2 Department of Science and Technology for Agriculture, Forestry, Nature and Energy (DAFNE), Tuscia University, Viterbo, Italy; 3 Department of Plant Sciences and Plant Pathology, Montana State University, Bozeman, Montana, United States of America; University of Exeter Medical School, United Kingdom

## Abstract

The *Pseudomonas syringae* complex is composed of numerous genetic lineages of strains from both agricultural and environmental habitats including habitats closely linked to the water cycle. The new insights from the discovery of this bacterial species in habitats outside of agricultural contexts *per se* have led to the revelation of a wide diversity of strains in this complex beyond what was known from agricultural contexts. Here, through Multi Locus Sequence Typing (MLST) of 216 strains, we identified 23 clades within 13 phylogroups among which the seven previously described *P. syringae* phylogroups were included. The phylogeny of the core genome of 29 strains representing nine phylogroups was similar to the phylogeny obtained with MLST thereby confirming the robustness of MLST-phylogroups. We show that phenotypic traits rarely provide a satisfactory means for classification of strains even if some combinations are highly probable in some phylogroups. We demonstrate that the citrate synthase (*ct*s) housekeeping gene can accurately predict the phylogenetic affiliation for more than 97% of strains tested. We propose a list of *cts* sequences to be used as a simple tool for quickly and precisely classifying new strains. Finally, our analysis leads to predictions about the diversity of *P. syringae* that is yet to be discovered. We present here an expandable framework mainly based on *cts* genetic analysis into which more diversity can be integrated.

## Introduction

*Pseudomonas syringae* was first reported as a plant pathogen of lilac by van Hall in 1902 [1]. Since its first description, *P. syringae* has become recognized as a phylogenetic complex of strains from terrestrial and aquatic habitats [2]. The classification of strains into the various sub-groups that constitute this complex has mirrored the historical trends in bacterial classification that were initially based on phenotypes (physiological and ecological characteristics) and then progressively were based on genotypes (DNA-DNA hybridization, phylogenetic analysis of housekeeping genes sequences) [3]. Commonly, seven phylogroups based on housekeeping gene phylogeny are recognized in the *P. syringae* complex [4] and some authors also include *P. cichorii* a closely related phytopathogenic species [5], [6]. These seven groups are more or less consistent with the species or genomospecies described based on DNA-DNA hybridization [7], [8] such as *P. viridiflava* [9] and *P. avellanae* [10] the latter recently re-defined with more accurate genomic analysis [11]. As for many bacterial pathogens, the affiliation of strains into pathovars is very common for the *P. syringae* group. Although the concept of pathovar is not related to phylogeny, pathovars are frequently used as an analytical framework for classifications based on physiological phenotypes [12], [13], MLST (Multi Locus Sequence Typing) phylogeny [14]–[16] or DNA-DNA hybridization [7]. More recently, strains of *P. syringae* were isolated from contexts where they were saprophytes in a range of environmental substrates. For these strains, the concept of pathovar had no apparent relevance, especially as they sometimes represented phylogroups not previously described among the strains isolated from diseased plants [2], [17]. These discoveries raise questions about how to classify these strains that have not been resolved in a standardized way.

In light of the growing diversity of what is being called *P. syringae* and of the lack of a guide for homogenous classification and naming of strains, we were led to examine the validity of the biochemical indicators and to attempt to clarify the situation. Here we present the results of genotypic and phenotypic characterization of 763 strains of *P. syringae* collected from a wide range of habitats in which this bacterium has been described up to date. These strains were selected to represent the full breadth of the genetic diversity in a collection of over 1600 strains of *P. syringae* for which some phylogenetic information was available. Through phylogenetic analyses based on 4 housekeeping genes we defined 23 clades within 13 phylogroups. Robustness of phylogroups was shown through core genome phylogeny on 29 strains representative of 9 of the 13 phylogroups. Phenotypic characterization on 763 strains illustrated that phenotypic traits provide only limited means for identification of strains at the clade or phylogroup level. A generalized linear model (GLM) procedure led to the identification of some highly probable significant combinations of phenotypes for eight phylogroups. We illustrate that the *cts* housekeeping gene alone can accurately predict the phylogenetic situation for most strains at the phylogroup and clade level. Overall, we describe the diversity of *P. syringae* and the utility of the data-base as a tool for classifying strains. Our analysis permits predictions about the diversity of *P. syringae* beyond what has been discovered and hence it provides a framework for future studies of the ecology of this bacterium.

## Materials and Methods

### Bacterial strains

The total of 836 strains used in this study is listed in Table S1 with their origin, alternative names and characteristics. Most strains were taken from a collection of over 7000 strains of *P. syringae* maintained at INRA in Montfavet (France). This collection was initiated in about 1995 and consists of strains collected from crops and from different environmental habitats via isolation on modified medium B of King (KBC) [18], [19]. For strains that were isolated from Grand Tetons National Park they were collected in accordance with permit number GRTE-2007-SCI-0023 issued to the corresponding author by the US Department of Interior, National Park Service, Office of Science and Resource Management of Grand Teton National Park. This declaration of sampling in accordance with this permit has also been made in the publication where these strains were originally reported [2]. For all other sites, no specific permissions were required. We selected 763 *P. syringae* strains isolated from fresh water and epilithic biofilms (56%), snowpack (16%), plants (11%), precipitation (9%), and litter (8%) that represented the range of genetic diversity of *P. syringae* and according to a procedure described in the supplementary information files (see Text S1). Some strains from crops not classified as quarantine organisms and providing reference phylogenetic information were kindly provided by colleagues or obtained from public collections.

### Genomic and phylogenetic analysis

MLST analysis was performed by sequencing four housekeeping genes: *cts* (encoding citrate synthase), *gapA* (glyceraldehyde-3-phosphate dehydrogenase A), *rpoD* (RNA polymerase sigma^70^ factor) and *gyrB* (gyrase B), using the Morris MLST schema of the Plant Associated and Environmental Microbes Database (PAMDB, http://genome.ppws.vt.edu/cgi-bin/MLST/home.pl) in combination with *gap*A and *gyr*B of the Hwang PAMDB schema [16], [20]. For each locus, sequences were extracted from GenBank and PAMDB, aligned with the *P. syringae* sequences by using DAMBE software version 5 [21] and were cut to the same size (1859 bp for the concatenated sequences). In order to clarify the phylogenetic position of strain LzW4 isolated from Antarctica and misclassified as *P. syringae* [22], housekeeping gene sequences were obtained from its genome. The concatenated sequences were used to construct the phylogeny with maximum likelihood and Bayesian methods by using the PHYLIP package version 3.6 (http://evolution.genetics.washington.edu/phylip.html) and Mr. Bayes version 3.1.2, respectively [23]. For maximum likelihood analysis, consensus trees were created from 100 independent phylogenies. Bayesian trees were constructed by using 500,000 generations with a burn-in period of 250,000. All sequences and critical metadata of strains were deposited in the PAMDB data base [24]. Genetic distances among the strains were determined with the Kimura 2-parameter model, with a gamma correction of 1, by using the PHYLIP package. For delimitation of phylogroups, the distance used as a criterion was chosen to allow delineation of the seven previously-described phylogroups of *P. syringae*. For delineation of clades, we used the threshold value of 2.3% as well as the tree structure as previously described [2].

For strains not included in the MLST analysis (see Text S1), phylogenetic affiliation was determined based solely on their partial *cts* sequences (409 bp). We first validated this method on the set of 216 MLST-typed strains (see Text S1 for details). We determined that the partial *gapA* and *cts* sequences are the most efficient sequences for phylogroup delimitation (Table S2, S3, S4, S5 and S6). However, the *cts* being largely used in previous studies [2], [20] it was selected to classify the remaining strains. This allowed us to determine the *cts* distance thresholds of 4.0% for phylogroup and 1.8% for clade affiliations (Table S6). These threshold values were used to classify the remaining 614 strains using the distance matrix of *cts* sequences including the set of the 216 strains.

Phylogenetic analysis of partial core genomes (sequences of 107 genes) was also performed. Core genomes were extracted from 29 *P. syringae* genomes (Table S1) as described previously [25]. Alignment of the core genome was made by using DAMBE version 5 as described above and a Bayesian tree was built with Mr. Bayes. Accession numbers of all sequences are reported in Table S1.

### Rarefaction curves

Rarefaction curves were constructed by randomly sampling a set of 830 individuals representing 13 different phylogroups or 23 different clades in the same proportions as delimited by their assignment to clades and phylogroups as described above. Random samples were drawn 830 times from the set of individuals and the average cumulative numbers of clades or phylogroups observed for each draw were calculated with R software version 2.9.1 with an in-house program (The R Development Core Team, 2009) after 1,000 iterations of the succession of draws.

### Phenotypic characterization

Phenotypic tests (Table S1) included production of fluorescent pigments on KB and tests in the LOPAT scheme (levan production, presence of cytochrome *c* oxidase, induction of potato soft rot, presence of arginine dihydrolase and induction of a hypersensitive reaction (HR) on tobacco) were performed as described previously [17]. In addition, tests for aesculin degradation, acidification of sucrose, and utilization of D(-) tartrate as a sole carbon source were performed as previously described [26], [27]. Strains were also tested for ice nucleation activity (INA), production of syringomycin-like toxins, pathogenicity and level of aggressiveness (see details in Text S1). This test was also used as a proxy of the extent of host-range of pathogenic strains as demonstrated previously [17].

### Phenotypic statistical analysis

Frequencies of the different significant combinations of all 11 phenotypes among the 13 *P. syringae* phylogroups were compared with a generalized linear approach as described in [27]. For each combined phenotype value (positive or negative), the probability for a strain with this combination to be in a given phylogroup was assessed by fitting a generalized linear model (GLM; [28]) to the data. In this analysis, the phylogroup number was introduced as the binary response variable modelled with the Bernoulli distribution, the phenotypes were explanatory variables and the logistic function was used for the link function. Significance of observed frequencies was addressed as described by Monteil and co-workers [27] by comparing them with those expected under the null hypothesis. The null hypothesis was rejected for P<0.05.

## Results and Discussion

### Delimitation of the phylogroups and clades represented in the strain collection

**Figure 1:**
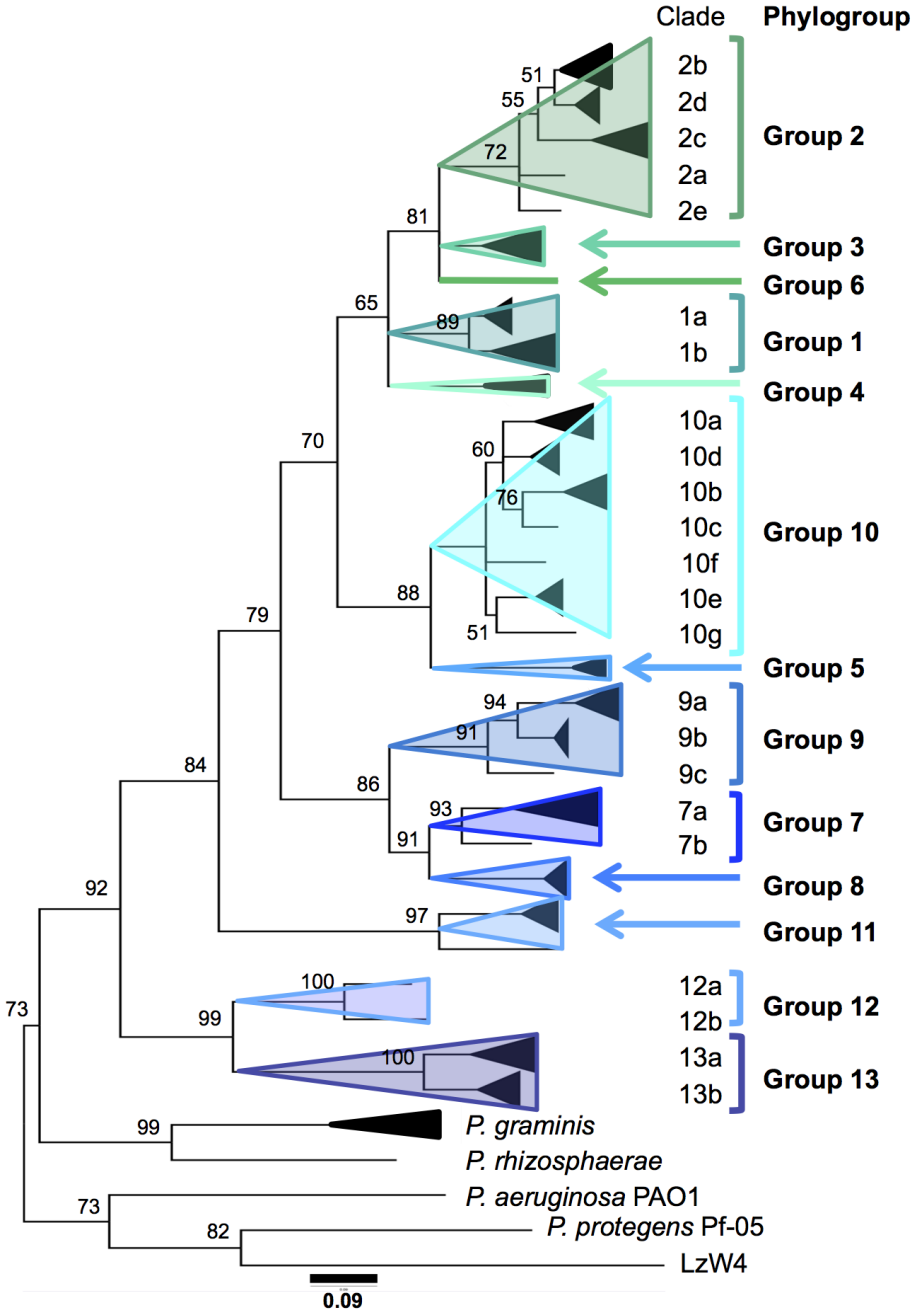
Bayesian tree constructed on the concatenated sequences *cts*, *gyrB*, *gapA* and *rpoD* of 216 *P. syringae* strains. Bootstrap values are showed at each node. Strain taxa were compressed and clade and phylogroup names are indicated (see the expanded tree with strain names in Fig. S1). Phylogroups from 1 to 7 were already reported in Parkinson et al. [4], phylogroup 8 in [30], phylogroups 9, 10, and 13 were described with other names by Morris and coworkers [2] (see Table S8 for name correspondence), phylogroup 11 corresponds to *P. cichorii* strains and phylogroup 12 was not described previously. The tree was rooted on *P. aeruginosa* PAO1.

The MLST analysis of the 216 *P. syringae* strains representing the maximum diversity of this group of bacteria revealed 13 groups composed of multiple sub-groups constituting 23 total clades (Fig. 1). Only seven phylogroups were subdivided into clades (Fig. 1). All strains analyzed had a genetic distance less than 5% with strains from their own phylogroup and more than 5% with strains outside their phylogroup with some exceptions (PsyCit7, CCV0213, CCV0567 and FMU107) (Table S7). The mean genetic distances within and outside phylogroups showed that they are relatively homogeneous and distinctly different from each other (Table 1).

**Table 1:** Mean genetic distances within (boldface values) and between phylogroups.

Number of strains	*P. syringae* Phylogroup	Mean genetic distance^a^												
		1	2	3	4	5	6	7	8	9	10	11	12	13
29	**1**	**0.02**												
84	**2**	0.10	**0.02**											
14	**3**	0.09	0.07	**0.02**										
5	**4**	0.10	0.10	0.09	**0.00**									
2	**5**	0.10	0.11	0.11	0.11	**0.00**								
1	**6**	0.10	0.09	0.08	0.11	0.11	**0.00**							
12	**7**	0.11	0.13	0.12	0.12	0.12	0.12	**0.03**						
4	**8**	0.13	0.14	0.13	0.14	0.13	0.13	0.07	**0.00**					
11	**9**	0.12	0.13	0.13	0.14	0.13	0.13	0.08	0.08	**0.02**				
38	**10**	0.11	0.11	0.11	0.11	0.07	0.12	0.12	0.14	0.13	**0.03**			
3	**11**	0.17	0.17	0.16	0.17	0.17	0.17	0.17	0.17	0.16	0.17	**0.06**		
2	**12**	0.16	0.16	0.16	0.17	0.16	0.16	0.18	0.19	0.17	0.17	0.19	**0.05**	
10	**13**	0.17	0.18	0.18	0.19	0.17	0.17	0.18	0.18	0.18	0.17	0.20	0.13	**0.03**
2	***P. graminis***	0.21	0.21	0.21	0.22	0.21	0.20	0.21	0.22	0.22	0.21	0.23	0.22	0.22
1	***P. rhizosphaerae***	0.21	0.21	0.21	0.21	0.22	0.23	0.20	0.19	0.22	0.22	0.23	0.22	0.23
1	***P. protegens***	0.20	0.19	0.19	0.20	0.19	0.19	0.19	0.18	0.20	0.20	0.20	0.21	0.21
1	***P. aeruginosa***	0.34	0.22	0.35	0.35	0.35	0.35	0.33	0.34	0.34	0.35	0.35	0.35	0.35

a Distances based on 1854 bp sequences of four housekeeping genes are those used for constructing the MLST tree of the set of 216 strains in Figure 1.

The phylogroups were robust independent of the phylogenetic model used to construct the phylogeny. Delimitation of phylogroups was determined by accounting for both tree branches (Fig. S1a) and genetic distances among the strains (Table S7). A genetic distance between concatenated sequences of less than 5% defined clearly the seven previously described phylogroups: phylogroups 1, 2 and 3 [29], phylogroup 4 [15], phylogroup 5 [16] and phylogroups 6 and 7 [4]. We used the same distance threshold of 5% for the delimitations of additional phylogroups (Table S7, Fig. S1a). Names previously attributed to well-known phylogroups (1 to 7) or clades, such as 2a, 2c or the recently named phylogroup 8 [30], were maintained to avoid confusion. Correspondences between the names proposed here and those of reference strains named as species, pathovars and genomo-species are indicated in Table S8. Among the reference strains used to construct the tree, *P. graminis* and *P. rhizosphaerae* strains were included to better delimit the *P. syringae* monophyletic group. These species are among the closest species outside the boundary of the *P. syringae* complex [6]. Three reference strains of *P. cichorii* formed a monophyletic clade clearly included in the *P. syringae* group (Fig. S1a). The *P. cichorii* CFBP 4407 strain had a distance higher than 5% from the two others (Table S7) but we nevertheless compressed all three strains in the *P. cichorii* phylogroup 11 (Fig. 1) since we considered that the diversity and phylogeny of *P. cichorii* is not well characterized and needs further investigation. This group represents the oxidase positive lineage of the *P. syringae* group of strains. Finally, our analysis reveals that strain LzW4 isolated from Antarctica and previously named *P. syringae* [22] is most closely related to *P. protegens* Pf-5 and it is clearly outside the *P. syringae* complex (Fig. 1).

**Figure 2:**
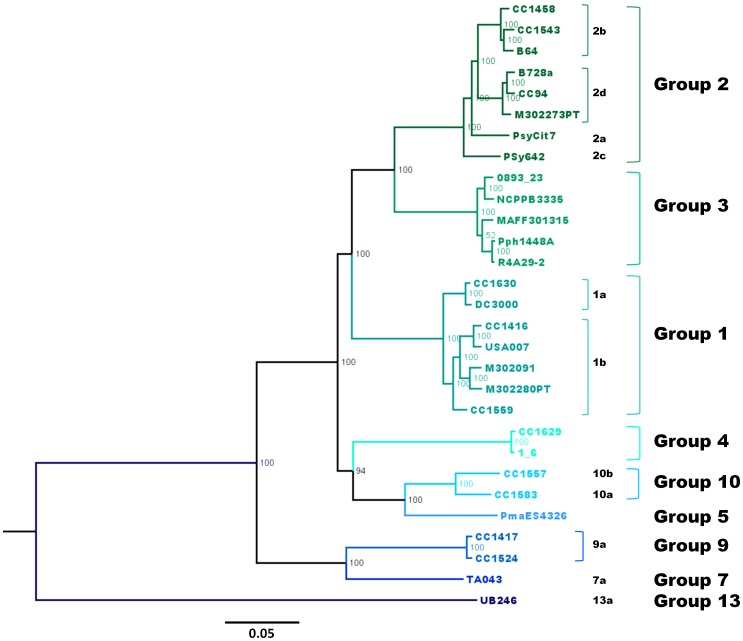
Bayesian phylogeny of the core genome of 29 *P. syringae* strains. An un-rooted tree was constructed on 107 open reading frames (64,000 bp) common to 29 *P. syringae* strains. Bootstrap values are indicated at each node and strain names are indicated at tree branches. Phylogroup and clade names are also indicated in the tree.

The phylogeny based on MLST of the 216 *P. syringae* strains is the framework we used to classify the remaining strains. To evaluate its robustness, we compared this phylogeny to that based on sequences of nearly whole core genomes of 29 strains representative of all the phylogroups except phylogroups 6, 8, 11 and 12 since no genomes were available for those phylogroups. The phylogeny based on 107 open reading frames (64,000 bp) illustrated in the unrooted tree in Fig. 2 showed the same phylogroup topology as the tree based on four housekeeping genes (Fig. 1). This result suggests that phylogeny at phylogroup level is robust enough to be represented by MLST analysis and that core genome analysis is not indispensable for studying the diversity of *P. syringae* and classifying strains within phylogroups. The robustness of phylogeny based on MLST was demonstrated for the *P. syringae* phylogroups 1 to 5 with seven genes independently [15]. Here we confirmed the robustness of phylogeny for all *P. syringae* phylogroups 1 to 5 and demonstrated it for phylogroups 7, 9, 10 and 13.

### Construction of a data base of 763 phenotyped strains of P. syringae classified into phylogroups and clades

To obtain the broadest range of information about the characteristics of the phylogroups and clades delimited here, we sought to classify the remaining 614 strains by using a reliable method that is simpler than MLST. With this aim, we used genetic distances and a tree constructed with only the *cts* housekeeping gene.

**Figure 3:**
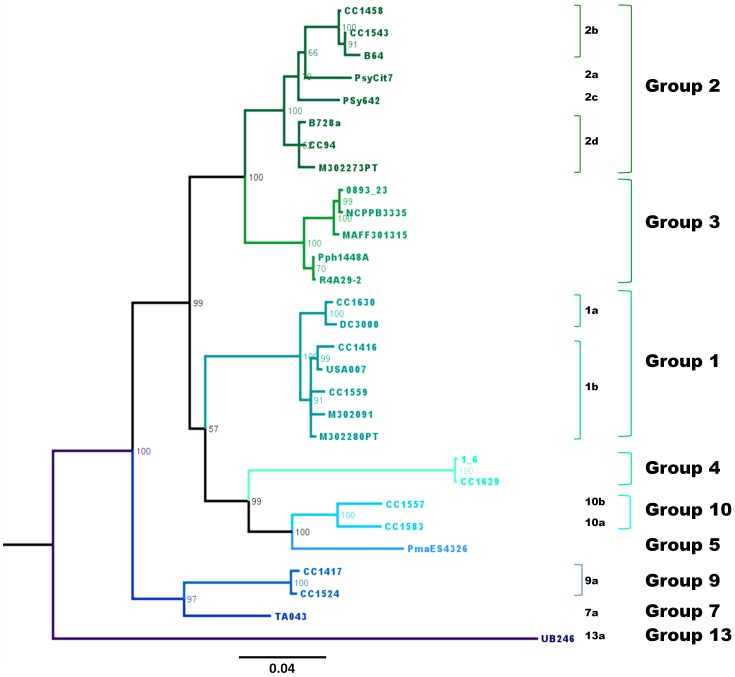
Bayesian phylogeny of the *cts* housekeeping gene of the 29 *P. syringae* strains used for core genome phylogeny. The phylogenetic un-rooted tree was made with the full-length *cts* gene (1290 bp) extracted from the genomes of the 29 *P. syringae* strains. Bootstrap values are indicated at each node and names of the strains at tree branches.

The *cts* gene was chosen as a *P. syringae* classification tool because, as previously described [15], it corresponds to one of the most reliable gene sequences among the genes used in MLST. It has the minimum number of recombinations and the most congruence among the trees constructed with housekeeping genes [15]. To reinforce these previous observations, we compared the tree based on the core genomes (Fig. 2) with that built on only *cts* gene and we showed that phylogeny at phylogroup level was consistent (Fig. 3). The classification of the 216 strains based on the *cts* gene sequence analysis validated our *P. syringae* classification tool with few exceptions (3/216). These exceptions show that some strains needed more than one housekeeping gene to be robustly classified (Table S6). We classified the 614 remaining strains without ambiguities except for 16 strains that were equidistant to phylogroup 1a and one strain of phylogroup 1b (Table S9). These strains were finally affiliated to phylogroup 1a based on their placement in the phylogenetic tree. On the total set of the 830 strains of *P. syringae*, we calculated that 97.6% strains were classified via the method proposed here. Finally, a total of 763 strains were classified into phylogroups and clades and characterized for their phenotypes. This constitutes a rather complete database useful for classification and characterization of strains that belong to the *P. syringae* complex (Table S1).

### Characteristics of the 13 phylogroups and associated clades

**Figure 4:**
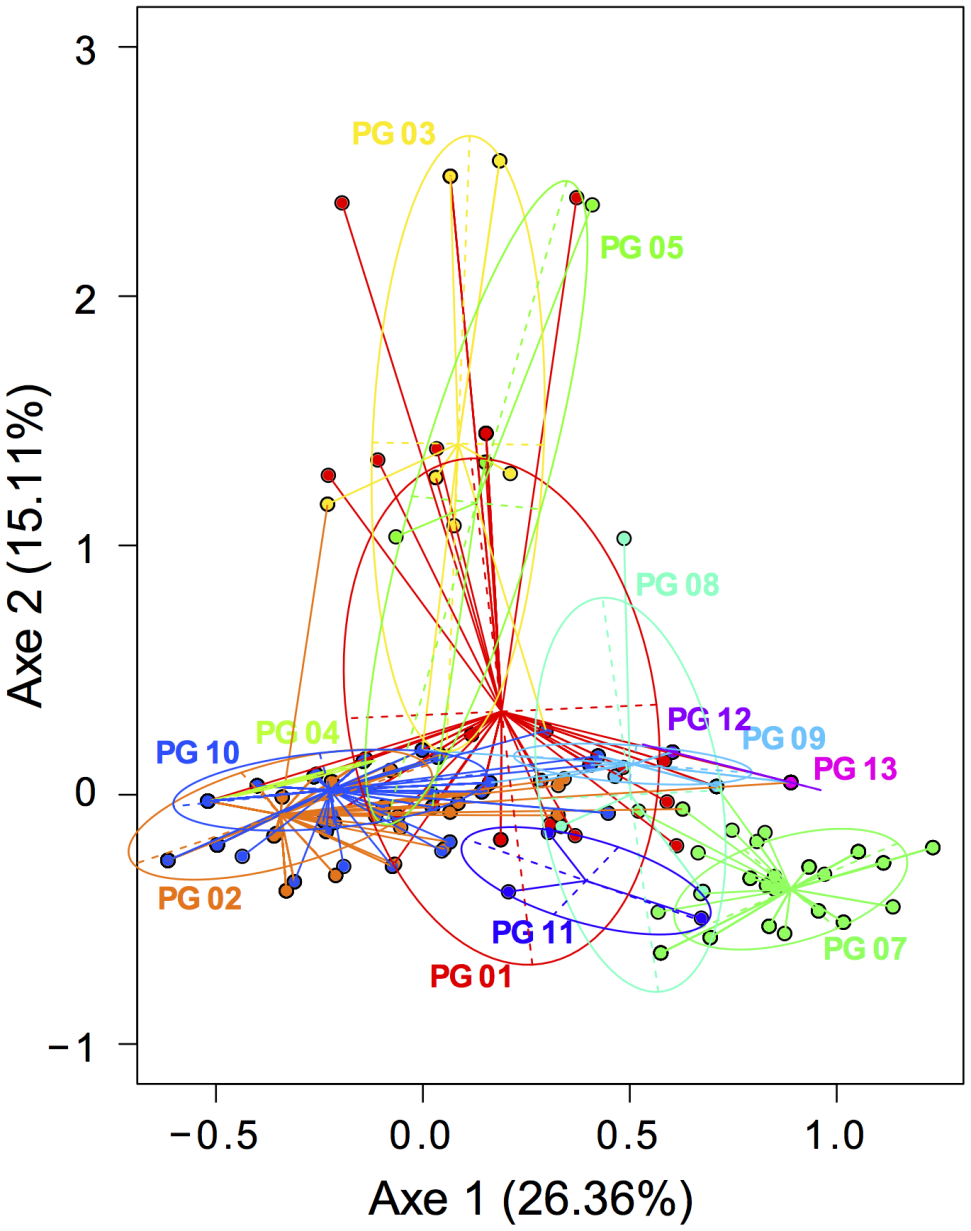
Phenotypic patterns associated with each of the 13 phylogroups of *P. syringae* revealed by a Multiple Correspondence Analysis (MCA). The analysis was based on 763 isolates and 11 phenotypes (see Table 2). Each color and each ellipse symbolize one phylogroup. A Monte-Carlo test (999 replicates) on a linear discriminant analysis confirmed that phenotypic dissimilarities were higher between phylogroups than within groups (*P*<0.001).

The results we provide for phylogroups clearly showed that phenotypes of strains are variable among and within phylogroups (Table 2). A multivariate correspondence analysis (MCA) followed by a discriminant analysis and a Monte-Carlo test showed that phenotypic variability was significantly higher (P<0.001) among phylogroups than within phylogroups as is expected (Fig. 4). Some phylogroups had unique phenotypic patterns (phylogroup 7), but some were very similar to each other (phylogroups 2, 4 and 10) (Fig. 4). Patterns of positive and negative responses were variable among phenotypes (see individual scatter plots in Fig. S2). To identify specific combinations of phenotypes for each phylogroups, we used a generalized linear approach. Combination of the 11 phenotypic traits for which the probability to belong to a given phylogroup is significantly higher than 0.8 are given in the Table S10 for phylogroups 01, 02, 07, 08, 09, 10, 11 and 13. A strain harboring one of these combinations has a high probability (>0.80) to belong to the corresponding phylogroup with a risk of error less than 0.05. The most probable combinations (>0.95) are those that could serve to classify strains according to their phylogroup. For phylogroups 03, 04, 05 and 12 no probable significant combination was found, likely due to the small number of strains (Table S10). Combinations not listed in Table S10 were not significant and could not be used for strain classification.

**Table 2:** Phenotypic characterization of 763 strains representing the genetic diversity in the *P. syringae* complex.

Phylogenetic affiliation	Number of strains	Levan +	Oxidase +	Potato soft rot	HR on tobacco+	Fluorescence on KB medium +	Aesculin degradation +	Sucrose utilization+	D(-) Tartrate utilization +	I NA^b^ +	Broad host-range toxin^c^ +	Pathogenicity^d^+	Avirulent^e^	Mean sdisease severity ≥2.0^f^
**Total**	**763**	**41**	**0**	**10**	**73**	**98**	**97**	**73**	**32**	**65**	**51**	**28**	**53**	**12**
**PG** a **01**	**88**	**39**	**0**	**0**	**92**	**85**	**92**	**91**	**58**	**33**	**2**	**16**	**60**	**1**
PG 01a	48	45	0	0	90	100	100	88	78	24	4	14	53	2
PG 01b	40	33	0	0	95	68	83	95	35	43	0	18	68	0
**PG 02**	**323**	**50**	**0**	**0**	**72**	**100**	**100**	**95**	**10**	**85**	**90**	**43**	**41**	**25**
PG 02a	3	0	0	0	100	100	100	100	33	67	67	33	33	0
PG 02b	140	67	0	0	98	100	99	95	4	90	87	44	30	19
PG 02c	91	7	0	0	7	100	100	93	9	68	96	0	89	0
PG 02d	87	70	0	0	100	100	100	98	20	93	91	89	6	62
PG 02e	2	0	0	0	50	100	100	100	0	100	100	0	100	0
**PG 03**	**10**	**70**	**0**	**0**	**80**	**70**	**20**	**90**	**0**	**20**	**0**	**0**	**90**	**0**
**PG 04**	**6**	**83**	**0**	**0**	**100**	**100**	**100**	**100**	**0**	**100**	**17**	**0**	**67**	**0**
**PG 05**	**4**	**50**	**0**	**0**	**75**	**75**	**25**	**75**	**25**	**50**	**0**	**25**	**50**	**0**
**PG 07**	**81**	**46**	**0**	**98**	**62**	**100**	**100**	**9**	**96**	**33**	**0**	**47**	**37**	**11**
PG 07a	79	46	0	97	61	100	100	9	96	33	0	48	37	11
PG 07b	2	50	0	100	100	100	100	0	100	50	0	0	50	0
**PG 08**	**5**	**80**	**0**	**40**	**100**	**100**	**80**	**0**	**100**	**0**	**100**	**0**	**80**	**0**
**PG 09**	**28**	**11**	**0**	**0**	**68**	**100**	**100**	**14**	**32**	**4**	**0**	**0**	**71**	**0**
PG 09a	23	9	0	0	65	100	100	13	26	4	0	0	70	0
PG 09b	4	25	0	0	75	100	100	25	50	0	0	0	75	0
PG 09c	1	0	0	0	100	100	100	0	100	0	0	0	100	0
**PG 10**	**167**	**34**	**0**	**0**	**98**	**100**	**100**	**84**	**11**	**94**	**51**	**10**	**66**	**3**
PG 10a	71	8	0	0	97	100	100	82	23	94	1	7	80	1
PG 10b	77	58	0	0	100	100	100	86	3	94	91	1	62	0
PG 10c	1	0	0	0	100	100	100	0	0	100	0	0	100	0
PG 10d	8	13	0	0	100	100	100	75	0	100	75	50	25	13
PG 10e	8	50	0	0	88	100	100	100	0	100	100	75	13	38
PG 10f	1	100	0	0	100	100	100	100	100	100	100	0	100	0
PG 10g	1	0	0	0	0	100	100	100	0	0	0	0	100	0
**PG 11**	**3**	**0**	**100**	**0**	**67**	**100**	**100**	**0**	**33**	**0**	**100**	**67**	**33**	**0**
**PG 12**	**2**	**0**	**0**	**0**	**0**	**100**	**100**	**0**	**50**	**0**	**0**	**0**	**100**	**0**
PG 12a	1	0	0	0	0	100	100	0	0	0	0	0	100	0
PG 12b	1	0	0	0	0	100	100	0	100	0	0	0	100	0
**PG 13**	**46**	**0**	**0**	**0**	**0**	**100**	**100**	**0**	**100**	**0**	**0**	**0**	**78**	**0**
PG 13a	44	0	0	0	0	100	100	0	100	0	0	0	77	0
PG 13b	2	0	0	0	0	100	100	0	100	0	0	0	100	0

Values are the percent of *P. syringae* strains giving positive reactions for the different phenotypes. Arginine dihydrolase production was negative for all strains.

a PG =  *P. syringae* phylogroup.

b INA = ice nucleation activity of at least 10^6^ cells at >−8°C.

c Production of a broad host range toxin was evaluated with the test habitually used to reveal syringomycin-like toxins based on the capacity to produce an inhibition zone of growth of *Geotricum candidum*.

d Strains were considered to be pathogenic on the cantaloupe indicator plant if at least half (6/12) of the seedlings showed compatible reactions.

e Avirulent strains did not induce any disease reaction on cantaloupe seedlings.

f Frequency of strains for which the mean disease severity on cantaloupe seedlings was ≥2.0.

However, some noteworthy results emerged from analysis of phenotypic traits: i) arginine dihydrolase was absent in all strains without exception; since this phenotype was used as criterion for elimination during isolation steps, positive strains could have been missed; ii) as expected, the only phylogroup that was oxidase-positive was phylogroup 11 containing the *P. cichorii* strains; the possibility that oxidase positive strains in the *P. syringae* complex from other phylogroups were discarded during isolation could not be excluded; iii) the production of fluorescent pigments and degradation of aesculin are the phenotypes that were positive for all strains in all phylogroups except for phylogroup 1, 3 and 5; iv) 65% of strains were ice nucleation active and the absence of this activity was observed for all strains in phylogroups 8, 11, 12 and 13; v) HR on tobacco was positive for 73% of the strains; vi) only 28% of the strains were pathogenic on cantaloupe seedlings; and vii) production of toxins inhibiting *Geotricum candidum* was frequent for strains of phylogroup 2, but also for phylogroup 8, 10 and 11. For phylogroup 2 and recently in the phylogroup 10 [31] genes for syringomycin toxins have been described and are likely to be involved in the toxicity observed here. But the mechanisms for the production of this toxin remain to be investigated for phylogroups 1, 4, 8 and 11.

Phenotypic and genotypic traits of each phylogroup follow:

***P. syringae*** **phylogroup 1** contains many strains from diseased plants but also from numerous environmental habitats and substrates [2] (Table S1). Phylogroup 1 consists of two clades described by other authors [8], [11]. Strains in clade 1a include *P. s.* pv. *tomato* (Table S1 and S8). Clade 1b includes *P. avellanae* and *P. s.* pv. *actinidiae*, respectively the causal agents of bacterial canker of hazelnut and kiwifruit (Table S1 and S8). Strains in this clade, as well as strains in phylogroup 3, contain a catechol operon regrouping genes for degradation of aromatic compounds [32]. Strains in clades 1a and 1b were similar in terms of their phenotypic variability, except that ca. 32% of the latter did not produce fluorescent pigment on KB and 17% did not degrade aesculin. All the strains that did not degrade aesculin carried the genes for degradation of aromatic compounds ([32]. Genomic studies have shown that among all phylogroups, strains of phylogroup 1 have the greatest number of Type Three Effector (T3E) genes coding for virulence determinants [25], [33] (Table 3). More recently, Monteil and coworkers [34] demonstrated that strains closely related to the tomato speck pathogen *P. s.* pv. *tomato* isolated from snowpack and streams harbor the T3E genes found in epidemic strains. Expression of most T3E genes are driven by the HrpL sigma factor that also regulates non-T3E genes associated with virulence. All the genes regulated by the HrpL sigma factor are called HrpL regulons [35]. Consistent with this observation, *P. s.* pv. *tomato* DC3000 in clade 1a has the greatest number of HrpL regulons described to date [35].

**Table 3:** Highlight features of the phylogroups of *Pseudomonas syringae* based on the profiles of strains characterized to date.

Phylogroup	Known habitats^a^	T3SS organization^b^	n° of T3SS effectors	n° of HrpL regulons	Broad host range toxins^d^	Soft rot induction	Putative host range^e^	INA^f^ at ≥− 8°C
**1**	Ubiquitous	Canonical	+++	+++	+	−	Narrow	++
**2**	Ubiquitous	Canonical & atypical	+	++	+++	−	Wide for some clades	+++
**3**	Mostly plants	Canonical	++	++	−	−	Host specific or narrow	++
**4**	Ubiquitous	Canonical	++	+++	++	−	Host specific or narrow	+++
**5**	Plants	Canonical	++	++	−	−	Intermediate	++
**6**	Plants	Canonical	++	+	ND	ND	ND	ND
**7**	Ubiquitous	Canonical & atypical	++	+	−	+++	Wide	++
**8**	Ubiquitous	Canonical & atypical	++	ND	++	+++	Narrow	−
**9**	Environment	ND^c^	ND	+		−	Null or very narrow	+
**10**	Environment	Canonical	+	+	+++	−	Wide for some clades	+++
**11**	Ubiquitous	Atypical	ND	ND	++	−	Wide	−
**12**	Environment	ND	ND	ND	−	−	Null or very narrow	−
**13**	Environment	Atypical	++	ND	−	−	Null or very narrow	−

These features are a general summary of the phenotypic and genotypic profiles garnered in this study as well as in other reports cited throughout the text above.

a Representatives of some phylogroups have been found associated only with plants, or only in non-agricultural (environmental) habitats or are ubiquitously present in all or nearly all habitats investigated to date.

b Both canonical and non-canonical (atypical) T3SS are found in some phylogroups, but they have not been found to co-exist in the same strain.

c This property has not been described to date.

d The production of broad host range toxins is based on the results of antibiosis tests reported in this work.

e Aggressiveness on cantaloupe seedlings was used as a proxy for host range as described previously [17].

Here, host range concerns the number of plant species on which disease symptoms are caused. The description presented for each phylogroup is relative to the other phylogroups and is based on the results of our analyses here. The range of epiphytic plants that can be colonized asymptomatically can be much larger than the host range for disease.

f Ice nucleation activity.

***P. syringae*** **phylogroup 2** is the most ubiquitous phylogroup of *P. syringae* found in all habitats analyzed to date [2]. In this phylogroup, three subgroups had been described previously, 2a, 2b and 2c [36] (Table S8). Phylogroup 2 is in fact composed of five different clades all containing some non-plant derived strains: i) *P. syringae* clade 2a contains strain PsyCit7 isolated from an asymptomatic orange tree [37], a strain from rain and one from an irrigation basin; ii) *P. syringae* clade 2b includes the *P. syringae* pv. *syringae* type strain (CFBP 1392^T^), *P. s.* pv. *aptata*, *P. s.* pv. *atrofaciens* and many strains isolated from all environmental substrates; iii) *P. syringae* clade 2c is dominated by non-pathogenic *P. syringae* strains isolated from plants and environmental substrates having an atypical Type Three Secretion System (T3SS) similar to the T3SS of S-PAI *P. viridiflava* [36]. Many strains in this clade contain identical sequences of a bacteriophage unique to this clade [38]; iv) *P. syringae* clade 2d contains strain B728a and is closely related to clade 2b; and v) *P. syringae* clade 2e is presently represented by only two strains isolated from fresh water and snow (Table S1). Although phylogroup 2 contains some strains incapable of inducing HR on tobacco (mainly in the clade 2c), we confirmed that strains in this phylogroup are on average more aggressive on cantaloupe seedlings than strains in all other phylogroups (Table 1) [2]. They are among the most consistently ice nucleation active (85% of strains) and most of them (90%) produce a syringomycin-like toxin (Table 2). Up until the recent characterization of strains in phylogroup 10 (described below) [31], the genomes of phylogroup 2 strains had been considered to carry the fewest T3E genes among all phylogroups [25] (Table 3). In parallel, they have numerous genes for phytotoxins such as syringolin, syringopeptin and syringomycin [25]. Strain B728a in clade 2d was recently reported to carry the fewest HrpL regulons [35].

***P. syringae*** **phylogroup 3**. The previously reported descriptions of this group that included many pathovars (Table S8) are not greatly influenced by our study because only very few strains in phylogroup 3 were isolated from environmental sources [39]. This result could be partly due to a bias in the isolation method. Strains of phylogroup 3 tend to grow more slowly on KB media than strains of phylogroup 2 for example, and hence they could have been missed. This group contains pathogens of woody plants (*P. savastanoi* pv. *savastanoi*, *P. s.* pvs. *aesculi*, *and mori*) that have been found to carry genes for the degradation of aromatic compounds [40], [41], but also contains pathogens of other types of host plants such as soybean and French bean (*P. s.* pvs. *glycinea* and *phaseolicola*). Among the notable phenotypic traits, incapacity to degrade aesculin and to produce fluorescent pigment on KB medium were frequent, similar to the properties observed in clade 1b.Phylogroup 3 strains were rarely ice nucleation active (20%) and none produced a syringomycin-like toxin (Table 2).

***P. syringae*** **phylogroup 4**. Few strains have been described in this phylogroup to date but seven pathovars have been reported (Table S8). Strains were isolated from diverse sources including cropped and wild plants (mostly monocotyledonous), rain, snowpack and plant litter (Table S1). As for phylogroup 3, strains from environmental substrates were rare and this could have resulted from a sampling bias. In contrast to other phylogroups, all strains in phylogroup 4 were ice-nucleation active. Interestingly, although these strains have been rarely detected in the environment, they were nevertheless among the highly ice-nucleation active strains from clouds on the Puy de Dôme in France [42]. Concerning the T3E repertoire within this phylogroup, two new T3SS genes, *hopBH1* and *hopBI1*, were recently described in the strains 1_6 (pathogenic on rice), CC1513 (from healthy wild *Hutchinsia alpine*) and CC1629 (from cropped *Avena sativa*). Strain 1_6 has been reported to have the greatest number of HrpL regulons (T3E and non-T3E) in the *P. syringae* complex [35].

***P. syringae*** **phylogroup 5** is represented by only five strains here (Table S1). Phylogroup 5, that includes strains pathogenic on diverse plants such as *Cannabis sativa*, *Brassicacae*, or coriander (Table S8), was not found to be abundant in the environment. Phenotypes within this group were highly variable in spite of the limited number of strains (Table 2).

***P. syringae*** **phylogroup 6** at present contains only strains isolated from diseased crops, including *Asteraceae* (*P. s. pv. tagetis*) and papaya (*P. caricapapayae*) in particular (Table S8). Only one strain, *P. s.* pv. *helianthi* (CFBP 2067) was included in this study. Diversity of this phylogroup in the environmental context still needs to be investigated.

***P. syringae*** **phylogroup 7** represents most of the strains called *P. viridiflava* in previous studies, as well as two *P. syringae* pathovars (*P. s.* pv. *ribicola* and pv. *primulae*) (Table S8) and many strains from a wide range of environmental reservoirs (Table S1). Almost all strains from phylogroup 7 are capable of causing soft rot to potato slices and to display phase variation [30]. This latter behavior has a considerable impact on several phenotypes including soft rot of potato and pathogenicity. Phylogroup 7 consists of two clades, 7a containing most of the strains (Table S1). Strains from phylogroup 7 harbor one non-canonical T3SS that resembles the one found in clade 2c [30], [43] (Table 3).

***P. syringae*** **phylogroup 8** was recently described [30] and contains strains that could also be called *P. viridiflava*. These strains share numerous characteristics with those in phylogroup 7, including phase variation [30]. However, they all produce a toxin in the bioassays with *G. candidum*. Due to the absence of syringomycin genes (unpublished data), this toxicity could be the result of the production of an antimycotic peptide such as ecomycin identified in *P. viridiflava* [44].

***P. syringae*** **phylogroup 9** strains have only been reported in aquatic habitats (Table S1). They did not produce syringomycin-like toxin, and only 4% were ice nucleation active. Three clades were delineated, with no distinct phenotypic differences between them. Phylogroup 9 corresponds to the phylogroup previously named CC1524 [2] (Table S8). Analysis of the genome of strain CC1524 revealed that it harbored novel HrpL regulons that were not found in strains in the other phylogroups [35].

***P. syringae*** **phylogroup 10** strains were the second most abundant in the collection analyzed in this study. They were exclusively from environmental reservoirs outside of areas cultivated for agriculture (Tables S1 and S8). However, almost all strains in phylogroup 10 (98%) induce HR on tobacco, 94% are ice nucleation active and 10% are pathogenic on cantaloupe seedlings. Genes for syringomycin-like toxins were found in the two full genome sequences available for this group (CC1583 and CC1557) and 51% of strains produced a syringomycin-like toxin in the bioassay. Seven clades were delimited, three of them corresponding to previously described clades “USA102” (10a), “TA0003” (10b), “USA032” (10e) (Table S8) [2] and three others containing only one strain (Table S1). The genomes of strains in phylogroup 10 have been recently reported to have the fewest T3E genes among all the strains in the *P. syringae* complex for which the T3SS has been characterized [31]. As mentioned above, this characteristic had been previously attributed to phylogroup 2 before genomes of strains of phylogroup 10 were available [25]. Finally, phylogroup 10 is quite comparable to phylogroup 2 in terms of its ubiquity, phenotypes, and number of T3E (Table 3). Since these two phylogroups are phylogenetically distant, convergent evolution could have shaped their behavior through horizontal gene transfer and other evolutionary processes linked to environmental pressures.

***P. syringae*** **phylogroup 11** is formed by strains that were classified in the *P. cichorii* species. This species was distinguished originally from *P. syringae* because of its cytochrome *c* oxidase, absent from *P. syringae* [18]. In this study we did not isolate strains of this phylogroup because only oxidase-negative isolates were retained in our basic isolation process. The inclusion of the *P. cichorii* lineage in the *P. syringae* complex was already proposed on the basis of phylogeny of housekeeping genes [6]. Strains belonging to phylogroup 11 are reported to be pathogenic on many crops such as lettuce or tomato [45]. *P. cichorii* has also been isolated from irrigation water [46]. The ecology of this phylogroup in environmental habitats and its diversity in a non-agricultural context remain to be explored. The three strains of phylogroup 11 tested here produced a toxin in the bioassays with *G. candidum*. It is likely that this toxicity is not due to syringomycin-like toxins as already shown by Hu et al. [47]. Interestingly the cytochrome *c* oxidase operon present in *P. cichorii* was also found in the genome of phylogroup 7 strains (CC1582 and TA043) but in none of the other phylogroups (unpublished data). However, all phylogroup 7 strains are negative for the phenotypic oxidase test. Furthermore, the single component-T3SS (S-PAI) of phylogroup 7 strains is evolutionarily related to the T3SS of phylogroup 11 strains [30] and we hypothesize that the S-PAI configuration represents the most ancient form of T3SS in the *P. syringae* complex.

***P. syringae*** **phylogroup 12** is composed of two strains (GAW0112 and GAW0113) isolated from water in an irrigation canal. Each of these strains represents a distinct clade. Overall, phylogroup 12 strains resemble phylogroup 13 strains (see below) in terms of the phenotypes characterized here. The diversity of this phylogroup still needs to be investigated.

***P. syringae*** **phylogroup 13**. Numerous strains isolated from non-plant substrates were found to be affiliated to phylogroup 13 (Table S1), previously called group UB246 [2] (Table S8). A recent study showed the existence of phylogroup 13 strains in wild alpine plants [48], suggesting that phylogroups such as this one are more wide-spread than our work indicates. The phenotypes of strains in phylogroup 13 were relatively homogenous (Table 2). Two clades were delimited, 13a containing most of the strains. A phylogenetic analysis of the *hrcC* T3SS gene showed that the T3SS of phylogroup 13 is more related to the *P. viridiflava* S-PAI than to the canonical T3SS of other *P. syringae* phylogroups (Table 3) [30].

### Tools and guidelines for classifying strains in the *P. syringae* complex

The use of phenotypic traits to classify strains in the *P. syringae* complex is sometimes the only option for identification, in particular for small diagnostic laboratories or when resources are limited. Using combinations of phenotypic tests according to Table S10 could be a means to attempt to classify strains of *P. syringae* with the known and traditional limitations of the use of phenotypic methods. A more accurate method of identification is often needed for specific epidemics or for diseases caused by a diversity of *P. syringae* strains in some cases from multiple phylogroups [34], [49]–[52]. Phenotypic criteria used during the screening of strains can markedly limit the diversity revealed in ecological or epidemiological studies. The only traits used in the initial selection of strains in this study were the absence of arginine dihydrolase and cytochrome *c* oxidase and the capacity to grow on KBC medium, which contains cephalexin and boric acid as selective agents. These traits might have limited the diversity of isolated strains. However, without a selective medium it would have been impossible to reveal the presence of *P. syringae* in most environmental reservoirs where it can constitute a mere 0.1% or less of the total bacterial population [20], [27]. Isolation of *P. syringae* with classical microbiological methods still remains a technique of choice for studying a bacterium with relatively very low abundance in most of the substrates it inhabits, diseased plants being the principal exception. Production of fluorescent pigments has been very useful to differentiate colonies of *P. syringae* and the occurrence of non-fluorescent strains complicates comprehensive ecological studies. The presence of the operon for pyoverdin production in the genomes of non-fluorescent strains (such as all *P. s.* pv. *actinidiae*) and recent successes to express fluorescence in these strains on different media [53] suggest the possibility to improve differential media for production of fluorescent pigment.

Phylogroup 11, the “oxidase-positive lineage” of *P. syringae*, is an exception that requires another isolation procedure since they can grow on KB medium, but not all strains are able to grow on KBC medium. Moreover, the positive oxidase test cannot distinguish strains of phylogroup 11 from other ubiquitous fluorescent pseudomonads related to *P. fluorescens* [54].

The most innovative part of the classification tool we propose here is the comprehensive data base (Table S1) that we provide to the scientific community. The originality of this data base is the information about strains isolated outside of agricultural contexts and from many different substrates beyond plants. It provides a broader vision of the perimeter of the *P. syringae* group and leads to the elaboration of new hypotheses on ecology of this group and disease emergence [32], [34]. The method we used to establish the framework for *P. syringae* classification is based on standard MLST, recognized as being reliable for many bacterial species and first proposed in 1998 by Maiden and colleagues [55]. It was validated in the *P. syringae* group firstly with 7 housekeeping genes [15] then with the four genes, *cts*, *gpaA*, *gyrB* and *rpoD* [16] that we have used here for MLST. We demonstrate that for the purpose of classification of strains one housekeeping gene can be sufficient. Such a simplification has been already proposed with the *rpoD* gene of *P. syringae* by Parkinson et al. [4] based on analysis of only the pathotype strain of each pathovar (phylogroups 1 to 7). Here we have chosen the *cts* gene on the basis of a comparison between the four single gene sequences of the strains that belong to all the 13 phylogroups (Table S6). We validated the MLST and *cts* phylogeny by whole core-genome phylogeny (Fig. 1, 2 and 3), showing that a low number of housekeeping genes can accurately assign *P. syringae* strains to phylogroups. The genomic approach however showed that within-group relationships can be misleading compared to genomic phylogeny [11], [56]. In our study, we proposed a subgroup classification in clades within some phylogroups to reflect the existing sub-group classification of phylogroup 2 [36]. These subgrouping in clades should be considered with caution in particular for evolutionary studies [56]. The fine relationship between two closely related strains that belong to one phylogroup could be investigated with fingerprinting techniques such as rep-PCR [57]. But these techniques can be complicated to realize and are mostly used to follow clonal lines during epidemics [57]. Comprehensive genomic comparisons remain the most reliable to discern two closely related strains and to understand their evolutionary relationships [56].

Our results illustrate that, at present, the most precise and efficient means to classify strains in the *P. syringae* complex is to compare the sequences of their *cts* genes to that of the strains used in this study. The robustness of the analysis and accuracy of *cts* were shown with full-length sequence data (1290 bp) from genomes (Fig. 2, Fig. 3), but also with only its partial sequence (409 bp) (Table S6). For routine phylogenetic analysis, only partial *cts* sequences (409 bp) could be used. We propose a list of reference strains labelled with their phylogenetic affiliation, together with their partial *cts* sequences in a table (Table S11) as well as in a FASTA file (File S1). These strains represent all phylogroups and clades identified in this study. For each clade and each phylogroup the most distant strains from the set of 216 *P. syringae* characterized by MLST were selected for *cts* sequence analysis. These sequences have also been deposited in GenBank (accession numbers indicated in Table S11). The procedure of classification consists of the following steps: 1) alignment of the partial *cts* gene sequence of the strain to be identified with those in Table S11; 2) analysis of tree branches and of the matrix of pair-wise distances to find the phylogroup and clade with which it is most similar and 3) assignment to the phylogroup or clade if the following criteria are met: <4% difference in the sequences for assignment to a phylogroup, and <1.8% difference for assignment to a clade, keeping in mind certain caveats. Due to possible recombination in housekeeping genes, affiliation to the clade level can be uncertain and especially when differences are near or >1.8%. Furthermore, as suggested below, a few new phylogroups and many new clades of *P. syringae* are yet to be found. Uncertainties in classification can be addressed by sequencing additional housekeeping genes and performing phylogenetic analyses based on sequences of multiple genes as recommended previously [8]. The data base that we describe here could provide a useful framework for characterization of new biodiversity.

### New diversity to anticipate in the *P. syringae* complex

**Figure 5:**
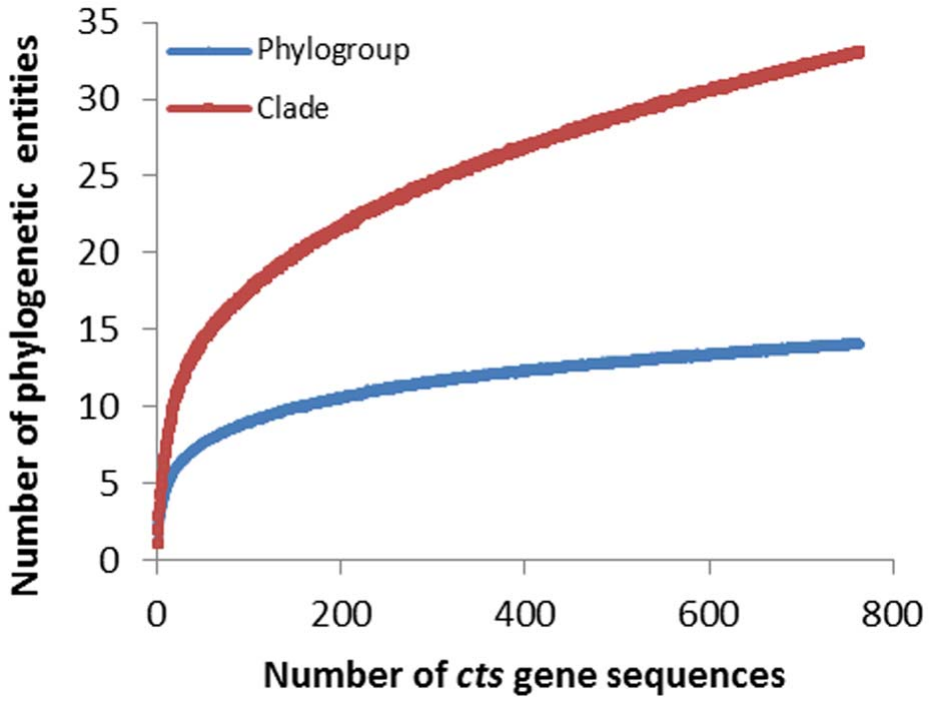
Rarefaction analysis of the *cts* gene sequences from strains of *P. syringae* at both phylogroup (blue curve) and clade (red curve) levels.

The diversity we described here is likely to be only a fraction of the entire *P. syringae* diversity. The rarefaction analysis suggests that the number of phylogroups revealed in this study is near its maximum but the number of clades is much smaller than the maximum in the total *P. syringae* meta-population (Fig. 5). Hence, descriptions of many new clades and some new phylogroups should be anticipated. A preponderance of our strain sources are from France. Exploration of additional ecosystems in other geographic locations is likely to increase the probability of discovery of even more genetic diversity of *P. syringae* than we predict here. Populations of endophytic *P. syringae* in native bitter cress (*Cardamine cordifolia*) growing in a subalpine context in Colorado at an elevation above 3,000 m have recently been characterized [48]. These endophytes were highly diverse belonging to phylogroups 1, 5, 7, 10, and 13 and to three putative new phylogroups. New diversity of *P. syringae* might also be found in association with hosts other than plants (algae, insects, fungi,) or in marine or other more extreme habitats. For example, strain CFII64 isolated from the highly contaminated Clark Fork river in Montana in a study of tolerance to cadmium exposure (http://www.ebi.ac.uk/ena/data/view/GCA_000416235) is closely related to phylogroup 13. This illustrates the diversity that remains to be discovered in this bacterial group and the need for a consistent way to classify strains.

## Conclusions

We propose a clear and standard classification of 13 phylogroups and associated clades forming the *P. syringae* complex. This classification considers the ensemble of strains described to date and provides a comprehensive analysis of the phenotypic variation in these phylogroups and clades relative to traits that have commonly been used to identify *P. syringae*. We clearly illustrate that, although phenotypes provide important ecological information, all single phenotypic traits tested here other than absence of cytochrome *c* oxidase activity and arginine dihydrolase can be misleading as a means to classify strains in the *P. syringae* complex. In this light we describe a simple method to identify strains of *P. syringae* based on the sequence of a single housekeeping gene and provide the data base needed for this approach. As population genomics emerges [58], it is likely that similar general conclusions will be made about genetic heterogeneity within phylogroups and clades.

By clarifying the classification of strains from a wide range of habitats and describing the genotypic and phenotypic profiles of the different phylogroups, we reveal a fascinating diversity of strategies deployed within the *P. syringae* complex (Table 3). These phylogroups vary in the nature of their T3SS and its efficiency in inciting plant disease, the balance of effectors, HrpL regulons and toxins in their genomes, and in the production of enzymes to degrade cell walls, for example (Table 3). These phylogroups all are apparently capable of surviving and multiplying in some environments in sufficient quantities to be detected in isolation schemes. These contrasting profiles raise questions about the fundamental traits of *P. syringae* that are essential for its survival and fitness and which of these are important in the potential of this bacterium to emerge in new epidemics of plant disease.

## Supporting Information

Figure S1 Bayesian trees constructed on concatenated sequences (*cts*, *gyrB*, *gapA* and *rpoD*) (A) and on only the *cts* sequence (B) for 216 *P. syringae* strains. Names of the strains were indicated at tree branches and trees were rooted on PAO1 and Pf-5.
(XLSX)

Figure S2 Scatter plots of the Multiple Correspondence Analysis outputs representing each phenotype. Each plot displays the categories for each phenotype and each dot represents an isolate.
(XLSX)

Table S1 Strain information, phenotypic tests, and *cts* sequences of the 836 strains used in this study. Phylogroup affiliation was assigned for each strain.
(XLSX)

Table S2 Matrix of genetic distances calculated for partial sequences of the *cts* gene of a set of 216 *P. syringae* strains. This set represents the maximum phylogenetic diversity of this group of bacteria and strains were previously classified in the MLST analysis. For each strain in a column, the minimum distance is highlighted in yellow. The distance thresholds for phylogroup and clade affiliations are respectively 0.04 and 0.018. Discrepancies between *cts* and MLST analysis are shown in pink.
(XLSX)

Table S3 Matrix of genetic distances calculated for partial sequences of the *gapA* gene of a set of 216 *P. syringae* strains. This set represents the maximum phylogenetic diversity of this group of bacteria and strains were previously classified in the MLST analysis. For each strain in a column, the minimum distance is highlighted in yellow. The distance thresholds for phylogroup and clade affiliations are respectively 0.06 and 0.029. Discrepancies between *cts* and MLST analysis are shown in pink.
(XLSX)

Table S4 Matrix of genetic distances calculated for partial sequences of the *gyrB* gene of a set of 216 *P. syringae* strains. This set represents the maximum phylogenetic diversity of this group of bacteria and strains were previously classified in the MLST analysis. For each strain in a column, the minimum distance is highlighted in yellow. The distance thresholds for phylogroup and clade affiliations are respectively 0.052 and 0.029. Discrepancies between *cts* and MLST analysis are shown in pink.
(XLSX)

Table S5 Matrix of genetic distances calculated for partial sequence of the *rpoD* gene of the 216 *P. syringae* strains. This set represents the maximum phylogenetic diversity of this group of bacteria and strains were previously classified in the MLST analysis. For each strain in a column, the minimum distance is highlighted in yellow. The distance thresholds for phylogroup and clade affiliations are respectively 0.053 and 0.019. Discrepancies between *cts* and MLST analysis are shown in pink.
(XLSX)

Table S6 Discrepancies between single gene (*cts*, *gapA*, *gyrB*, *rpoD*) and MLST analyses. Classification of a set of 216 *P. syringae* strains representing the maximum phylogenetic diversity of this group of bacteria was used. Affiliations of strains were based on distance matrixes (see Tables S2 to S5). Only strains with misidentification are reported.
(XLSX)

Table S7 Matrix of the genetic distances calculated in the MLST analysis between 216 *P. syringae* strains. This set of bacteria represents the maximum phylogenetic diversity of this group of bacteria, and the distance was calculated for the concatenated partial sequences of the *cts*, *gapA*, *gyrB* and *rpoD* genes.
(XLSX)

Table S8 Correspondence between *P. syringae* phylogroups and clades with other classification schemes (pathovar, species/genomo-species) described previously.
(XLSX)

Table S9 Matrix of genetic distances calculated for partial sequences of the cts genes of the 614 *P. syringae* strains to be classified and the set of 216 strains. This set represents the maximum phylogenetic diversity of this group of bacteria and strains were previously classified in the MLST analysis. For each strain in a column, the minimum distance is highlighted in yellow. The distance thresholds for phylogroup and clade affiliations are respectively 0.04 and 0.018. Ambiguous classification between *cts* and MLST analyses are shown in pink.
(XLSX)

Table S10 List of phenotype combinations having a significant probability superior to 0.8 to appear in a given phylogroup. For example, a strain that has the phenotype [fluorescence positive, oxidase negative, aesculine positive, levan positive, sucrose positive, potato soft rot negative, D(-) tartrate positive, HR positive, INA negative, pathogenicity on cantaloupe negative, syryngomycin production negative] has a probability between 0.80 and 0.85 to belong to the *P. syringae* phylogroup 01 with a probability of error of 0.05.
(XLSX)

Table S11 List of the 64 reference strains and their *cts* sequences representative of all *P. syringae* phylogroups and clades. This database is proposed for a rapid identification and classification of *P. syringae* strains. The neighbor joining tree for the 64 *cts* sequences is presented.
(XLSX)

File S1 Fasta file of the partial *cts* sequences of 68 reference strains. This file was designed for classifying putative *P. syringae* strains among the 13 *P. syringae* phylogroups trough a phylogenetic analysis. The phylogroup membership of a strain appears after its name.
(DOCX)

Text S1 Supplementary Information. Selection of *P. syringae* strains. Validation of the *cts* gene as a *P. syringae* tool classification. Characterization of ice nucleation activity. Characterization of syringomycin-like toxin production. Characterization of pathogenicity and aggressiveness of *P. syringae* strains.
(DOCX)
