# Supplementary material for: A User's Guide to a Data Base of the Diversity of Pseudomonas syringae and Its Application to Classifying Strains in This Phylogenetic Complex
Source: PLoS One. 2014 Sep 3;9(9):e105547. doi: 10.1371/journal.pone.0105547 (PMC4153583; doi:10.1371/journal.pone.0105547)
Supplement: File S1 — Fasta file of the partial cts sequences of 68 reference strains. This file was designed for classifying putative P. syringae strains among the 13 P. syringae phylogroups trough a phylogenetic analysis. The phylogroup membership of a strain appears after its name. (DOCX) [file pone.0105547.s014.docx]

**File S1. Fasta file of the partial *cts* sequences of 68 reference strains**. This file, was designed for classifying putative *P. syringae* strains among the 13 *P. syringae* phylogroups trough a phylogenetic analysis. The phylogroup membership of a strain appears after its name.

>T1_PG01a

CACCGGCCGCTTCACATTTGACCCTGGCTTC-ATGTCCACGGCCTC-TTGCGAGTCGAAGATCACCTACATTGATGGTGACAACGGAATTCTGCTGCACCGCGGCTACCCGATCGAACAACTGGCCGAGCAGTCCGATTACCTCGAGA-CCTGCTACCTGCTGCTCAACGGCGAGCTGCCAACCGCCGAACAGAAGGCCCAGTTCGTGGCCGTGGTCAAGAACCACACGATGGTTCACGAACAACTCAAGACCTTCTTCAACGGCTTTCGCCGTGACGCCCACCCGATGGCCGTCATGTGCGGTGTAGTTGGCGCCCTGTCGGCGTTCTACCACGATTCGCTGGACATCAATAACCCGCAGCACCGCGAAATTTCGGCTGTACGCCTGGTCGCCAAG-ATGCCGACCCTGGCA

>DC3000_PG01a

CACCGGCCGCTTCACATTTGACCCTGGCTTC-ATGTCCACGGCCTC-TTGCGAGTCGAAGATCACCTACATTGATGGTGACAACGGAATTCTGCTGCACCGCGGCTACCCGATCGAACAACTGGCCGAGCAGTCCGATTACCTCGAGA-CCTGCTACCTGTTGCTCAACGGCGAGCTGCCAACCGCCGAACAGAAAGCCCAGTTCGTGGCCGTGGTCAAGAACCACACGATGGTTCACGAACAACTCAAGACCTTCTTCAACGGCTTTCGCCGTGACGCCCACCCGATGGCCGTCATGTGCGGTGTAGTCGGCGCCCTGTCGGCGTTCTACCACGATTCGCTGGACATCAATAACCCGCAGCACCGCGAAATTTCGGCTGTACGCCTGGTCGCCAAG-ATGCCGACCCTGGCA

>CC1416_PG01b

CACCGGCCGCTTCACATTTGACCCTGGTTTC-ATGTCCACGGCCTC-TTGCGAGTCGAAGATCACCTACATTGACGGTGACAACGGAATTCTGCTGCACCGCGGCTACCCGATCGAACAACTGGCCGAGCAGTCCGATTACCTCGAGA-CCTGCTACCTGTTGCTCAACGGCGAGCTGCCAACTGCCGAACAGAAAGCCCAGTTCGTGGCCGTGGTCAAGAACCACACGATGGTTCACGAACAACTCAAGACCTTCTTCAACGGTTTTCGCCGTGACGCCCACCCGATGGCCGTCATGTGCGGTGTAGTCGGCGCGCTGTCGGCGTTCTACCACGATTCGCTCGACATCAATAACCCGCAGCACCGCGAAATTTCGGCTGTACGCTTGGTCGCCAAG-ATGCCGACCCTGGCA

>CC1427_PG01b

CACCGGCCGCTTCACATTTGACCCTGGTTTC-ATGTCCACGGCCTC-TTGCGAGTCGAAGATCACCTACATCGATGGTGACAACGGAATTCTGCTGCACCGCGGCTACCCGATCGAACAACTGGCCGAGCAGTCCGATTACCTCGAGA-CCTGTTACCTGTTGCTCAACGGCGAGCTGCCAACCGCCGAACAGAAAGCCCAGTTCGTGGCCGTGGTCAAGAACCACACGATGGTTCACGAACAACTCAAGACCTTCTTCAACGGCTTTCGCCGTGACGCCCATCCGATGGCCGTCATGTGCGGTGTAGTCGGCGCCCTGTCGGCGTTCTACCACGATTCGCTCGACATCAATAACCCGCAGCATCGCGAAATTTCGGCTGTACGCCTGGTCGCCAAA-ATGCCAACCCTGGCA

>CC1559_PG01b

CACCGGCCGCTTCACATTTGACCCTGGTTTC-ATGTCCACGGCCTC-TTGCGAGTCGAAGATCACCTACATCGATGGTGACAACGGAATTCTGCTGCACCGCGGCTACCCGATCGAACAACTGGCCGAGCAGTCCGATTACCTCGAGA-CCTGCTACCTGTTGCTCAACGGCGAGCTGCCAACCGCCGAACAGAAAGCCCAGTTCGTGGCCGTGGTCAAGAACCACACAATGGTTCACGAACAACTCAAGACTTTCTTCAACGGCTTTCGCCGTGACGCCCACCCGATGGCCGTCATGTGCGGTGTAGTCGGCGCCCTGTCGGCGTTCTACCACGATTCGCTGGACATCAATAACCCGCAGCACCGCGAAATTTCGGCTGTACGCCTGGTCGCCAAG-ATGCCGACCCTGGCA

>CSZ0761_PG01b

CACCGGCCGCTTCACATTTGACCCTGGTTTC-ATGTCCACGGCCTC-TTGCGAGTCGAAGATCACCTACATCGATGGTGACAACGGAATTCTGCTGCACCGCGGCTACCCGATCGAACAACTGGCCGAGCAGTCCGATTACCTCGAGA-CCTGCTACCTGTTGCTCAACGGCGAGCTGCCAACCGCCGAACAGAAAGCCCAGTTCGTGGCCGTGGTCAAGAACCACACAATGGTTCACGAACAACTCAAGACCTTCTTCAACGGCTTTCGCCGTGACGCCCACCCGATGGCCGTCATGTGCGGTGTAGTCGGCGCCCTGTCGGCGTTCTACCACGATTCGCTCGACATCAATAACCCGCAGCACCGCGAAATTTCGGCTGTACGCCTGGTCGCCAAG-ATGCCGACCCTGGCA

>M302091_PG01b

CACCGGCCGCTTCACATTTGACCCTGGTTTC-ATGTCCACGGCCTC-TTGCGAGTCGAAGATCACCTACATCGATGGTGACAACGGAATTCTGCTGCACCGCGGCTACCCGATCGAACAACTGGCCGAGCAGTCCGATTATCTCGAGA-CCTGCTACCTGTTGCTCAACGGCGAGCTGCCAACCGCCGAACAGAAAGCCCAGTTCGTGGCCGTGGTCAAGAACCATACGATGGTTCACGAACAACTCAAGACCTTCTTCAACGGCTTTCGCCGTGACGCCCACCCGATGGCCGTCATGTGCGGTGTAGTCGGCGCCCTGTCGGCGTTCTACCACGATTCGCTGGACATCAATAACCCGCAGCACCGCGAAATTTCGGCTGTACGCCTGGTCGCCAAG-ATGCCGACACTGGCA

>PavBPIC631_PG01b

CACCGGCCGCTTCACATTTGACCCTGGTTTC-ATGTCCACGGCCTC-TTGCGAGTCGAAGATCACCTACATTGACGGTGACAACGGAATTCTGCTGCACCGCGGCTACCCTATCGAACAACTGGCCGAGCAGTCCGATTACCTCGAGA-CCTGCTACCTGTTGCTCAACGGCGAGCTGCCAACCGCCGAACAGAAAGCCCAGTTCGTGGCCGTGGTCAAGAACCACACGATGGTTCACGAACAACTCAAGACCTTCTTCAACGGCTTTCGCCGTGACGCCCACCCGATGGCCGTCATGTGCGGTGTAGTCGGCGCGCTGTCGGCGTTCTACCACGATTCGCTCGACATCAATAACCCGCAGCACCGCGAAATTTCGGCTGTACGCCTGGTCGCCAAG-ATGCCGACCCTGGCA

>PsyCit7_PG02a

CACCGGCCGCTTCACATTTGACCCTGGTTTC-ATGTCCACGGCCTC-TTGCGATTCGAAGATCACCTACATCGATGGTGATAATGGCATTCTGCTACACCGCGGCTACCCGATCGAACAACTGGCCGAGCAGTCCGACTACCTCGAGA-CCTGCTACCTGCTGCTCAACGGTGAACTGCCCACCGCCGAGCAGAAAGCCCAGTTCGTGGCCGTGGTCAAGAACCACACGATGGTTCACGAACAACTCAAGACCTTCTTCAACGGTTTCCGCCGTGACGCCCACCCGATGGCCGTCATGTGTGGCGTAGTCGGCGCCCTGTCGGCGTTCTACCACGACTCGCTGGATATCAATAACCCGCAGCACCGCGAGATTTCCGCAGTCCGTCTGGTCGCCAAG-ATGCCGACCTTGGCA

>H5E1_PG02b

CACCGGCCGCTTCACATTTGACCCTGGTTTC-ATGTCCACGGCCTC-TTGCGATTCGAAGATCACCTACATCGATGGTGATAATGGCATTCTGCTACACCGCGGCTACCCGATCGAACAGCTGGCCGAGCAGTCCGACTACCTCGAAA-CCTGCTACCTGCTACTCAACGGTGAACTGCCTACCGCCGAGCAGAAAGCCCAGTTCGTGGCCGTGGTCAAGAACCACACGATGGTTCACGAACAGCTCAAGACCTTTTTCAACGGTTTCCGCCGCGACGCCCACCCGATGGCCGTCATGTGTGGCGTAGTCGGCGCCCTGTCAGCGTTCTATCACGACTCGCTGGACATCAATAACCCGCAGCACCGCGAGATTTCTGCGATCCGCCTGGTCGCCAAG-ATGCCGACCCTGGCA

>M301072PT_PG02b

CACCGGCCGCTTCACATTTGACCCTGGTTTC-ATGTCCACGGCCTC-TTGCGATTCGAAGATCACCTACATCGATGGTGACAATGGCATTCTGCTACACCGTGGCTACCCGATCGAACAACTGGCCGAGCAGTCCGACTATCTCGAAA-CCTGCTACCTGCTGCTCAACGGTGAACTGCCCACCGCCGAGCAGAAAGCCCAGTTCGTGGCCGTGGTCAAGAACCACACGATGGTTCACGAACAGCTCAAGACCTTTTTCAACGGTTTCCGCCGCGACGCCCACCCGATGGCCGTCATGTGTGGCGTAGTCGGCGCCCTGTCAGCGTTCTACCACGACTCACTGGACATCAATAACCCGCAGCATCGCGAGATTTCTGCGGTCCGCCTGGTCGCCAAA-ATGCCGACCCTGGCA

>CC457_PG02b

CACCGGCCGCTTCACATTTGACCCTGGTTTC-ATGTCCACGGCCTC-TTGCGATTCGAAGATCACCTACATCGATGGTGATAATGGCATTCTGCTACACCGCGGCTACCCGATCGAACAACTGGCCGAGCAGTCCGACTATCTCGAAA-CCTGCTACCTGCTGCTCAACGGTGAACTGCCCACCGCCGAGCAGAAAGCCCAGTTCGTGGCCGTGGTCAAGAACCACACGATGGTTCACGAACAGCTCAAGACCTTTTTCAACGGTTTCCGCCGCGACGCCCACCCGATGGCCGTCATGTGTGGCGTAGTCGGCGCCCTGTCAGCGTTCTACCACGACTCACTGGACATCAATAACCCGCAGCACCGCGAGATTTCTGCGGTCCGCCTGGTCGCCAAA-ATGCCGACCCTGGCA

>PavIsaPave013_PG02b

CACCGGCCGCTTCACATTTGACCCTGGTTTC-ATGTCCACGGCCTC-TTGCGATTCGAAGATCACCTACATCGATGGTGATAATGGCATTCTGCTACACCGCGGCTACCCGATCGAACAACTGGCCGAGCAGTCCGACTACCTCGAAA-CCTGCTACCTGCTGCTCAACGGTGAACTGCCCACCGCCGAGCAGAAAGCCCAGTTCGTGGCCGTGGTCAAGAACCACACGATGGTTCACGAACAGCTCAAGACTTTTTTCAACGGTTTCCGCCGCGACGCCCACCCGATGGCCGTCATGTGTGGCGTAGTCGGCGCCCTGTCAGCGTTCTACCACGACTCGCTGGACATCAATAACCCGCAGCACCGCGAGATTTCCGCGGTCCGCCTGGTCGCCAAG-ATGCCGACCCTGGCA

>508_PG02c

CACCGGCCGCTTCACATTTGACCCTGGCTTC-ATGTCCACGGCCTC-TTGCGATTCGAAGATCACCTACATCGACGGTGACAATGGCATTCTGCTGCACCGCGGCTATCCGATCGAACAACTGGCCGAGCAGTCCGACTACCTGGAAA-CCTGCTACCTGCTGCTCAACGGCGAACTGCCCACCGCCGAGCAGAAAGCCCAGTTCGTGGCCGTGGTCAAGAACCACACGATGGTTCACGAACAGCTCAAGACCTTTTTCAACGGTTTCCGCCGTGACGCCCACCCGATGGCCGTCATGTGTGGTGTAGTCGGCGCCCTGTCAGCGTTCTACCACGACTCCCTGGACATCAATAACCCGCAGCACCGCGAGATTTCCGCGGTCCGCCTGGTTGCCAAG-ATGCCGACCCTGGCA

>SZ0045_PG02c

CACCGGCCGCTTCACATTTGACCCTGGCTTC-ATGTCCACGGCCTC-TTGCGATTCGAAGATCACCTACATCGATGGTGATAATGGCATTCTGTTGCACCGCGGCTACCCGATCGAACAACTGGCCGAGCAGTCCGACTACCTGGAAA-CCTGCTACCTGCTGCTCAACGGCGAACTGCCCACCGCCGAGCAGAAAGCCCAGTTCGTGGCCGTGGTCAAGAACCACACGATGGTTCACGAACAGCTCAAGACCTTTTTCAACGGTTTCCGCCGTGACGCCCACCCGATGGCCGTCATGTGTGGTGTAGTCGGCGCCCTGTCAGCGTTCTACCACGACTCTCTGGACATCAATAACCCGCAGCATCGCGAGATTTCCGCGATCCGCCTGGTTGCCAAG-ATGCCGACCCTGGCA

>PSy642_PG02c

CACCGGCCGCTTCACATTTGACCCTGGCTTC-ATGTCCACGGCCTC-TTGCGATTCGAAGATCACCTACATCGATGGTGACAATGGCATTCTGCTACACCGCGGCTACCCGATCGAACAACTGGCCGAGCAGTCCGACTACCTGGAAA-CCTGCTACCTGCTGCTCAACGGCGAACTGCCCACCGCCGAGCAGAAAGCCCAGTTCGTTGCCGTGGTCAAGAACCACACGATGGTTCACGAACAGCTCAAGACCTTTTTCAACGGTTTCCGCCGTGACGCCCACCCGATGGCCGTCATGTGTGGTGTAGTCGGCGCCCTGTCAGCGTTCTACCACGACTCTCTGGACATCAATAACCCGCAGCACCGCGAGATTTCCGCGATCCGCCTGGTTGCCAAG-ATGCCGACCCTGGCA

>SZ0030_PG02c

CACCGGCCGCTTCACATTTGACCCTGGCTTC-ATGTCCACGGCCTC-TTGCGATTCGAAGATCACCTACATCGACGGTGACAATGGCATTCTGCTACACCGCGGCTACCCGATCGAACAACTGGCCGAGCAGTCCGACTACCTGGAAA-CCTGCTACCTGCTGCTCAACGGTGAACTGCCCACCGCCGAGCAGAAAGCCCAGTTCGTGGCCGTGGTCAAGAACCACACGATGGTTCACGAACAGCTCAAGACCTTTTTCAACGGTTTCCGCCGTGACGCCCACCCGATGGCCGTCATGTGTGGTGTAGTCGGCGCCCTGTCAGCGTTCTACCACGACTCTCTGGACATCAATAACCCGCAGCACCGCGAGATTTCCGCGATCCGCCTGGTTGCCAAG-ATGCCGACCCTTGCA

>CC1470_PG02d

CACCGGCCGCTTCACATTTGACCCTGGTTTC-ATGTCCACGGCGTC-TTGCGATTCGAAGATCACCTACATTGATGGTGACAATGGCATTCTGCTGCACCGCGGCTACCCGATCGAACAACTGGCAGAGCAGTCCGACTACCTCGAAA-CCTGCTACCTGCTGCTCAACGGCGAACTGCCCACCGCCGAGCAGAAAGCCCAGTTCGTGGCCGTGGTCAAGAACCACACGATGGTTCACGAGCAACTCAAGACCTTTTTCAACGGTTTCCGTCGCGACGCCCACCCGATGGCCGTCATGTGTGGCGTAGTCGGCGCCCTGTCAGCGTTCTATCACGACTCGCTGGACATCAATAACCCGCAGCACCGCGAGATTTCCGCGGTCCGCCTGGTCGCCAAG-ATGCCGACCCTGGCA

>USA011_PG02d

CACCGGCCGCTTCACATTTGACCCTGGTTTC-ATGTCCACGGCGTC-TTGCGATTCGAAGATCACCTACATCGATGGTGACAATGGCATTCTGCTGCACCGCGGCTACCCGATCGAACAACTGGCCGAGCAGTCCGACTACCTCGAAA-CCTGCTACCTGCTGCTCAACGGCGAACTGCCCACCGCCGAGCAGAAAGCCCAGTTCGTGGCCGTGGTCAAGAACCACACGATGGTTCACGAACAGCTCAAGACCTTTTTCAACGGTTTCCGCCGCGACGCCCACCCGATGGCCGTCATGTGTGGCGTGGTCGGTGCCCTGTCAGCGTTCTACCACGACTCGCTGGACATCAATAACCCGCAGCACCGCGAGATTTCCGCGGTACGCCTGGTCGCCAAG-ATGCCGACCCTGGCA

>B728a_PG02d

CACCGGCCGCTTCACATTTGACCCTGGTTTC-ATGTCCACGGCGTC-TTGCGATTCGAAGATCACCTACATCGATGGTGACAATGGCATTCTGCTGCACCGCGGCTACCCGATCGAACAACTGGCCGAGCAGTCCGACTACCTCGAAA-CCTGCTACCTGCTGCTCAACGGCGAACTGCCCACCGCCGAGCAGAAAGCCCAGTTCGTGGCCGTGGTCAAGAACCACACGATGGTTCACGAGCAACTCAAGACCTTTTTCAACGGTTTCCGTCGCGACGCCCACCCGATGGCCGTCATGTGTGGCGTAGTCGGTGCCCTGTCAGCGTTCTACCACGACTCGCTGGACATCAATAACCCGCAGCACCGCGAGATTTCCGCGGTACGCCTGGTCGCCAAG-ATGCCGACCCTGGCA

>CC0301_PG02d

CACCGGCCGCTTCACATTTGACCCTGGTTTC-ATGTCCACGGCGTC-TTGCGATTCGAAGATCACCTACATCGATGGTGACAATGGCATTCTGCTGCACCGCGGCTACCCGATCGAACAACTGGCCGAGCAGTCCGACTACCTCGAAA-CCTGCTACCTGCTGCTCAACGGCGAACTGCCCACCGCCGAGCAGAAAGCCCAGTTCGTGGCCGTGGTCAAGAACCACACGATGGTTCACGAGCAACTCAAGACCTTTTTCAACGGTTTCCGTCGCGACGCCCACCCGATGGCCGTCATGTGTGGCGTAGTCGGTGCCCTGTCAGCGTTCTACCACGACTCGCTGGACATCAATAACCCGCAGCACCGCGAGATTTCCGCGGTCCGCCTGGTCGCCAAG-ATGCCGACCCTGGCA

>USA0035_PG02e

CACCGGCCGCTTCACATTTGACCCTGGTTTC-ATGTCCACGGCCTC-TTGCGATTCGAAGATCACCTACATCGATGGTGACAATGGCATTCTGCTGCACCGCGGCTACCCGATCGAACAACTGGCCGAGCAGTCCGACTACCTCGAAA-CCTGCTATCTGCTGCTCAATGGCGAACTGCCCACCGCCGAGCAGAAAGCCCAGTTCGTGGCCGTGGTCAAGAACCACACGATGGTTCACGAACAACTCAAGACTTTCTTCAACGGTTTCCGCCGCGACGCCCACCCGATGGCCGTCATGTGTGGCGTAGTCGGCGCCCTGTCAGCGTTCTACCACGACTCGCTGGACATCAATAACCCGCAGCACCGCGAGATTTCCGCGGTCCGCCTGGTCGCCAAG-ATGCCGACCCTGGCA

>MAFF301315_PG03

CACCGGCCGCTTCACATTTGACCCTGGCTTC-ATGTCCACGGCCTC-TTGCGATTCGAAGATCACCTACATTGATGGTGACAATGGCATTCTGCTGCACCGCGGCTACCCGATCGAACAACTGGCCGAGCAGTCCGATTACCTCGAAA-CCAGCTACCTGCTGCTCAACGGCGAACTGCCGACCGCCGAACAGAAAGCCCAGTTCGTGGCCGTGGTCAAGAACCACACGATGGTTCACGAGCAACTCAAGACCTTCTTCAACGGCTTTCGTCGCGACGCCCACCCGATGGCCGTCATGTGCGGCGTAGTCGGTGCCCTGTCAGCGTTCTACCACGACTCGCTGGACATCAATAATCCGCAGCACCGCGAGATTTCCGCTGTACGCCTGGTGGCCAAG-ATGCCGACCCTGGCA

>MAFF302941_PG03

CACCGGCCGCTTCACATTTGACCCTGGCTTC-ATGTCCACGGCCTC-TTGCGATTCGAAGATCACCTACATTGATGGTGACAATGGCATTCTGCTGCACCGCGGCTACCCGATCGAACAACTGGCCGAGCAGTCCGATTACCTCGAAA-CCTGCTACCTGTTGCTCAACGGCGAACTGCCGACCGCCGAACAGAAAGCCCAGTTCGTGGCCGTGGTCAAGAACCACACGATGGTTCACGAGCAACTCAAGACCTTCTTCAACGGCTTTCGTCGCGACGCCCACCCGATGGCCGTCATGTGCGGCGTAGTCGGTGCCCTGTCAGCGTTTTACCACGACTCGCTGGACATCAATAACCCGCAGCACCGCGAGATTTCCGCTGTACGCCTGGTGGCCAAG-ATGCCGACCCTGGCA

>MAFF301020_PG03

CACCGGCCGCTTCACATTTGACCCTGGCTTC-ATGTCCACGGCCTC-TTGCGATTCGAAGATCACCTACATTGATGGTGACAATGGCATTCTGCTGCACCGCGGCTACCCGATCGAACAACTGGCCGAGCAGTCCGATTACCTCGAAA-CCTGCTACCTGCTGCTCAACGGCGAACTGCCGACCGCCGAACAGAAAGCCCAGTTCGTGGCCGTGGTCAAGAACCACACGATGGTTCACGAGCAACTCAAGACCTTCTTCAACGGCTTTCGTCGCGACGCCCACCCGATGGCCGTCATGTGCGGCGTAGTCGGTGCCCTGTCAGCGTTCTACCACGACTCGCTGGACATCAATAACCCGCAGCACCGCGAGATTTCCGCTGTACGCCTGGTGGCCAAG-ATGCCGACCCTGGCA

>LYR0002_PG03

CACCGGCCGCTTCACATTTGACCCTGGCTTC-ATGTCCACGGCCTC-TTGCGATTCGAAGATCACCTACATTGATGGTGACAATGGCATTCTGCTGCACCGCGGCTACCCGATCGAACAACTGGCCGAGCAGTCCGATTACCTCGAAA-CCTGCTACCTGCTGCTCAACGGCGAACTGCCGACCGCCGAACAGAAAGCCCAGTTCGTGGCCGTGGTCAAGAACCACACGATGGTTCACGAGCAACTCAAGACCTTCTTCAACGGCTTTCGTCGCGACGCCCACCCGATGGCCGTCATGTGCGGCGTAGTCGGTGCCCTGTCAGCGTTCTACCACGACTCGCTGGACATCAATAACCCGCAGCACCGCGAGATTTCCGCTGTACGCCTGGTGGCCAAG-ATGCCGACCCTGGCA

>Pph1448A_PG03

CACCGGCCGCTTCACATTTGACCCTGGCTTC-ATGTCCACGGCCTC-TTGCGATTCGAAGATCACCTACATTGATGGTGACAATGGCATTCTGCTGCACCGCGGCTACCCGATCGAACAACTGGCCGAGCAGTCCGATTACCTCGAAA-CCTGCTACCTGCTGCTCAACGGCGAACTGCCGACCGCCGAACAGAAAGCCCAGTTCGTGGCCGTGGTCAAGAACCACACGATGGTTCACGAGCAACTCAAGACCTTCTTCAACGGCTTTCGTCGCGACGCCCACCCGATGGCCGTCATGTGCGGCGTAGTCGGTGCCCTGTCAGCGTTCTACCACGACTCGCTGGACATCAATAACCCGCAGCACCGCGAGATTTCCGCTGTACGCCTGGTGGCCAAG-ATGCCGACCCTGGCA

>CC1513_PG04

CACCGGCCGCTTCACATTTGACCCTGGCTTC-ATGTCCACGGCATC-TTGCGAGTCGAAGATCACCTACATCGATGGTGACAATGGAATCCTGCTTCACCGCGGCTACCCTATCGAACAACTGGCCGAGCAGTCCGATTACCTCGAAA-CCTGTTACCTGCTGCTCAACGGCGAATTGCCCACCGCCGAACAGAAAGCCCAGTTTGTGGCCGTGGTCAAGAACCACACGATGGTTCACGAGCAACTCAAGACATTTTTCAACGGCTTCCGCCGCGATGCCCACCCGATGGCCGTCATGTGCGGCGTAGTCGGCGCCCTTTCTGCGTTCTATCACGACTCGCTGGACATCAATAACCCGCAGCACCGCGAAATTTCGGCAGTGCGCCTGGTAGCCAAG-ATGCCGACCCTGGCA

>CC1629_PG04

CACCGGCCGCTTCACATTTGACCCTGGCTTC-ATGTCCACGGCATC-TTGCGAGTCGAAGATCACCTACATCGATGGTGACAACGGAATCCTGCTTCACCGCGGCTACCCTATCGAACAACTGGCCGAGCAGTCCGATTACCTCGAAA-CCTGTTACCTGCTGCTCAACGGCGAATTGCCGACCGCCGAACAAAAAGCCCAGTTTGTGGCCGTGGTCAAGAACCACACGATGGTTCACGAGCAACTCAAGACATTTTTCAACGGCTTCCGCCGCGATGCCCACCCGATGGCCGTCATGTGCGGCGTAGTCGGCGCCCTTTCTGCGTTCTATCACGACTCGCTGGACATCAATAACCCGCAGCACCGCGAAATTTCGGCAGTGCGCCTGGTAGCCAAG-ATGCCGACCCTGGCA

>1_6_PG04

CACCGGCCGCTTCACATTTGACCCTGGCTTC-ATGTCCACGGCATC-TTGCGAGTCGAAGATCACCTACATCGATGGTGACAACGGAATCCTGCTTCACCGCGGCTACCCTATCGAACAACTGGCCGAGCAGTCCGATTACCTCGAAA-CCTGTTACCTGCTGCTCAACGGCGAATTGCCGACCGCCGAACAAAAAGCCCAGTTTGTGGCCGTGGTCAAGAACCACACGATGGTTCACGAGCAACTCAAGACATTTTTCAACGGCTTCCGCCGCGATGCCCACCCGATGGCCGTCATGTGCGGCGTAGTCGGCGCCCTTTCTGCGTTCTATCACGACTCGCTGGACATCAATAACCCGCAGCACCGCGAAATTTCGGCAGTGCGCCTGGTAGCCAAG-ATGCCGACCCTGGCA

>PmaES4326_PG05

CACCGGCCGCTTCACATTTGACCCTGGCTTC-ATGTCCACGGCATC-TTGCGAGTCGAAGATCACCTACATCGATGGTGATAACGGAATCCTGCTTCATCGCGGCTACCCGATCGAACAACTGGCCGAGCAGTCCGATTACCTGGAAA-CCTGCTACCTGCTGCTCAATGGCGAACTGCCAACTGCCGAGCAGAAAGCCCAGTTCGTCGCCGTGGTCAAGAACCACACGATGGTCCACGAACAGCTCAAGACCTTTTTCAACGGTTTTCGCCGTGATGCCCACCCGATGGCCGTCATGTGTGGCGTCGTCGGCGCCCTGTCGGCGTTCTATCACGACTCGCTGGACATCAATAACCCGCAACACCGCGAAATCTCCGCCGTGCGCCTGGTTGCCAAG-ATGCCGACCCTGGCA

>CFBP2067_PG06

CACCGGCCGCTTCACATTTGACCCTGGCTTC-ATGTCCACGGCCTC-TTGCGATTCGAAGATCACCTACATCGATGGTGACAATGGAATTCTGCTGCACCGCGGCTACCCGATCGAACAACTGGCCGAGCAGTCCGACTATCTCGAAA-CCTGCTATCTGCTGCTCAACGGCGAACTGCCCACCGCCGAACAGAAAGCCCAGTTCGTGGCCGTGGTCAAGAACCACACGATGGTTCACGAGCAACTCAAGACATTTTTCAACGGCTTTCGCCGCGACGCCCACCCGATGGCCGTCATGTGCGGCGTAGTCGGCGCTCTCTCGGCGTTCTACCACGACTCGCTGGACATCAATAATCCGCAGCACCGCGAAATTTCCGCCGTGCGCCTGGTTGCCAAG-ATGCCGACCCTGGCA

>CC1582_PG07a

CACCGGCCGCTTCACATTCGACCCTGGCTTC-ATGTCGACCGCCTC-TTGCGAGTCGAAGATCACCTACATCGATGGTGACAACGGAATCCTGCTGCACCGCGGCTACCCTATCGAACAACTGGCCCAGCACTCCGACTACCTGGAGA-CCTGCTACCTGCTGCTCAACGGTGAATTGCCAACCGCCGAGCAGAAAGCCCAGTTCGTCGCCGTGGTCAAGAACCACACGATGGTCCACGAACAGCTCAAGACCTTTTTCAACGGCTTCCGTCGCGACGCCCACCCGATGGCCGTCATGTGCGGCGTGGTCGGCGCGCTTTCAGCGTTCTACCACGACTCGCTGGACATCAATAATCCGCAGCACCGCGAGATTTCCGCCGTACGCCTGGTCGCCAAG-ATGCCGACCCTGGCC

>CMO0110_PG07a

CACCGGCCGCTTCACATTCGACCCTGGCTTC-ATGTCGACCGCCTC-TTGCGAGTCGAAGATCACCTACATCGATGGTGACAACGGAATCCTGCTGCACCGCGGCTACCCTATCGAACAACTGGCCCAGCACTCCGACTACCTGGAGA-CCTGCTACCTGCTGCTCAACGGCGAACTGCCGACCGCCGAGCAGAAAGCCCAGTTCGTCGCCGTGGTCAAGAACCACACGATGGTCCACGAACAGCTCAAGACCTTTTTCAACGGCTTCCGTCGCGACGCCCACCCGATGGCCGTCATGTGCGGAGTGGTCGGCGCCCTTTCAGCGTTCTACCACGACTCGCTGGACATCAATAATCCGCAGCACCGCGAGATTTCCGCCGTACGCCTGGTCGCCAAG-ATGCCGACCCTGGCC

>BS0002_PG07a

CACCGGCCGCTTCACATTCGACCCGGGCTTC-ATGTCGACCGCCTC-TTGCGAGTCGAAGATCACCTACATCGATGGTGACAACGGAATCCTGCTGCACCGCGGCTACCCTATCGAACAACTGGCCCAGCACTCCGACTACCTGGAGA-CCTGCTACCTGCTGCTCAACGGCGAATTGCCGACCGCCGAGCAGAAAGCCCAGTTCGTCGCCGTGGTCAAGAACCACACGATGGTCCACGAACAGCTCAAGACCTTTTTCAACGGCTTCCGTCGCGACGCCCACCCGATGGCCGTCATGTGCGGCGTGGTCGGCGCCCTTTCAGCGTTCTACCACGACTCGCTGGACATCAATAATCCGCAGCACCGCGAGATTTCCGCCGTACGCCTGGTCGCCAAG-ATGCCGACCCTGGCC

>TA0002_PG07a

CACCGGCCGCTTCACATTCGACCCTGGCTTC-ATGTCGACCGCCTC-TTGCGAGTCGAAGATCACCTACATCGATGGTGACAACGGAATCCTGCTGCACCGCGGCTACCCTATCGAACAACTGGCCCAGCACTCCGACTACCTGGAGA-CCTGCTACCTGCTGCTCAATGGCGAATTGCCGACCGCCGAGCAGAAAGCCCAGTTCGTCGCCGTGGTCAAGAACCACACGATGGTCCACGAACAGCTCAAGACCTTTTTCAACGGCTTCCGTCGCGACGCCCACCCGATGGCCGTCATGTGCGGCGTGGTCGGCGCGCTTTCAGCGTTCTACCACGACTCGCTGGACATCAATAATCCGCAGCACCGCGAGATTTCCGCCGTACGCCTGGTCGCCAAG-ATGCCGACCCTGGCC

>TA043_PG07a

CACCGGCCGCTTCACATTCGACCCTGGCTTC-ATGTCGACCGCCTC-TTGCGAGTCGAAGATCACCTACATCGATGGTGACAACGGAATCCTGCTGCACCGCGGCTACCCTATCGAACAACTGGCCCAGCACTCCGACTACCTGGAGA-CCTGCTACCTGCTGCTCAACGGCGAATTGCCGACCGCCGAGCAGAAAGCCCAGTTCGTCGCCGTGGTCAAGAACCACACGATGGTCCACGAACAGCTCAAGACCTTTTTCAACGGCTTCCGTCGCGACGCCCACCCGATGGCCGTCATGTGCGGCGTGGTCGGCGCGCTTTCAGCGTTTTACCACGACTCGCTGGACATCAATAATCCGCAGCACCGCGAGATTTCCGCCGTGCGCCTGGTCGCCAAG-ATGCCGACCCTGGCC

>FMU107_PG07b

CACCGGCCGCTTCACATTCGACCCGGGCTTC-ATGTCGACCGCCTC-TTGCGAGTCGAAGATCACCTACATCGATGGTGACAACGGAATCCTGCTGCACCGCGGCTACCCTATCGAACAACTGGCCCAGCACTCCGATTATCTGGAGA-CCTGCTACCTGCTGCTCAACGGCGAACTGCCAACCGCCGAGCAGAAAGCCCAGTTCGTCGCCGTGGTCAAGAACCACACGATGGTCCACGAACAGCTCAAGACCTTTTTCAACGGCTTCCGTCGCGACGCCCACCCGATGGCCGTCATGTGTGGCGTGGTCGGAGCCCTTTCAGCGTTCTACCACGACTCGCTGGACATCAATAATCCGCAGCATCGCGAGATTTCCGCCGTGCGCCTGGACGCCAAG-ATGCCGACCCTGGCA

>CMO0085_PG08

CACCGGCCGCTTCACATTCGACCCGGGCTTC-ATGTCGACCGCCTC-TTGCGAGTCGAAAATCACCTACATCGATGGTGACAACGGAATCCTGCTTCACCGCGGCTACCCTATCGAACAACTGGCCCAGCATTCCGACTACCTGGAAA-CCTGCTACCTGCTGCTCAACGGCGAATTGCCCACCGCCGAGCAGAAAGCCCAATTCGTTGCCGTGGTGAAAAACCACACGATGGTCCACGAACAGCTCAAGACCTTTTTCAACGGCTTTCGCCGTGACGCCCACCCGATGGCCGTCATGTGCGGCGTAGTCGGCGCATTGTCGGCGTTCTACCACGACTCGCTGGACATCAATAACCCGCAGCACCGCGAAATTTCCGCCGTACGTCTGGTCGCCAAG-ATGCCGACCCTGGCC

>GAW0203_PG08

CACCGGCCGCTTCACATTCGACCCGGGCTTC-ATGTCGACCGCCTC-TTGCGAGTCGAAAATCACCTACATCGATGGTGACAACGGAATCCTGCTTCACCGCGGCTACCCTATCGAACAACTGGCCCAGCATTCCGACTACCTGGAAA-CCTGCTACCTGCTGCTCAACGGCGAATTGCCCACCGCCGAGCAGAAAGCCCAATTCGTTGCCGTGGTGAAAAACCACACGATGGTCCACGAACAGCTCAAGACCTTTTTCAACGGCTTTCGCCGTGACGCCCACCCGATGGCCGTCATGTGCGGCGTAGTCGGCGCATTATCGGCGTTCTACCACGACTCGCTGGACATCAATAACCCGCAGCACCGCGAAATTTCCGCCGTACGCCTGGTCGCCAAG-ATGCCGACCCTGGCC

>CC1532_PG09a

CACCGGCCGCTTCACATTCGACCCTGGTTTC-ATGTCGACCGCCTC-TTGCGAGTCGAAAATCACCTACATTGATGGTGACAACGGAATTCTGCTGCACCGCGGCTACCCTATCGAGCAACTGGCGCAGCATTCCGATTATCTGGAAA-CCTGCTACCTGCTACTCAACGGCGAACTGCCGACCGCCGAGCAGAAGGCCCAGTTCGTCGCTGTGGTCAAGAACCACACGATGGTCCACGAACAGCTCAAGACCTTTTTCAACGGTTTCCGTCGCGACGCCCACCCGATGGCGGTCATGTGCGGCGTTGTAGGTGCCCTTTCAGCGTTCTACCACGACTCGCTGGACATCAATAACCCTCAGCACCGCGAAATTTCGGCAGTCCGCCTGGTCGCCAAG-ATGCCGACCCTGGCA

>CC1524_PG09a

CACCGGCCGCTTCACATTCGACCCTGGTTTC-ATGTCGACCGCCTC-TTGCGAGTCGAAAATCACCTACATTGATGGTGACAACGGAATTCTGCTGCACCGCGGCTACCCTATCGAGCAACTGGCGCAGCATTCCGATTATCTGGAAA-CCTGCTACCTGCTGCTCAACGGCGAACTGCCGACCGCCGAGCAGAAAGCCCAGTTCGTCGCTGTGGTCAAGAACCACACGATGGTCCACGAACAGCTCAAGACCTTTTTCAACGGCTTCCGTCGCGACGCCCACCCGATGGCGGTCATGTGCGGCGTTGTAGGTGCCCTTTCAGCGTTCTACCACGACTCGCTGGACATCAATAACCCTCAGCACCGCGAAATTTCGGCAGTCCGCCTGGTCGCCAAG-ATGCCGACCCTGGCA

>CC1417_PG09a

CACCGGCCGCTTCACATTCGACCCTGGTTTC-ATGTCGACCGCCTC-TTGCGAGTCGAAAATCACCTACATTGATGGTGACAACGGAATTCTGCTGCACCGCGGCTACCCTATCGAGCAACTGGCGCAGCATTCCGATTATCTGGAAA-CCTGCTACCTGCTGCTCAACGGCGAACTGCCGACCGCCGAGCAGAAAGCCCAGTTCGTCGCTGTGGTCAAGAACCACACGATGGTCCACGAACAGCTCAAGACCTTTTTCAACGGTTTCCGTCGCGACGCCCACCCGATGGCGGTCATGTGCGGCGTTGTAGGTGCCCTTTCAGCGTTCTACCACGACTCGCTGGACATCAATAACCCTCAGCACCGCGAAATTTCGGCAGTCCGCCTGGTCGCCAAG-ATGCCGACCCTGGCA

>TA0006_PG09b

CACCGGCCGCTTCACATTCGACCCTGGTTTC-ATGTCGACCGCCTC-TTGCGAGTCGAAGATCACCTACATCGATGGTGACAACGGAATCCTGCTGCACCGCGGCTACCCGATCGAACAACTGGCCCAGCATTCCGATTATCTGGAAA-CCTGCTACCTGCTGCTCAACGGCGAACTGCCAACCGCCGAGCAGAAAGCCCAGTTCGTCGCCGTGGTCAAGAACCACACGATGGTCCACGAACAGCTCAAGACCTTTTTCAACGGCTTCCGTCGCGACGCCCACCCGATGGCGGTCATGTGCGGCGTTGTAGGTGCCCTTTCAGCGTTCTACCACGACTCGCTGGACATCAATAACCCGCAGCACCGCGAAATTTCCGCAGTCCGCCTGGTCGCCAAG-ATGCCGACCCTGGCA

>CMW0020_PG09c

CACCGGCCGCTTCACATTCGACCCTGGTTTC-ATGTCGACCGCCTC-TTGCGAGTCGAAGATCACCTACATCGATGGTGACAACGGAATCCTGCTGCACCGCGGCTACCCGATCGAACAACTGGCCCAGCAGTCCGATTATCTGGAAA-CCTGCTACCTGCTGCTCAACGGTGAACTGCCTACCGCCGAGCAGAAAGCCCAGTTCGTCGCCGTGGTCAAGAACCACACGATGGTCCACGAACAGCTCAAGACCTTTTTCAACGGTTTCCGTCGCGACGCCCACCCAATGGCGGTCATGTGCGGCGTTGTAGGTGCCCTCTCAGCGTTCTACCACGACTCGCTGGACATCAATAACCCGCAGCACCGCGAAATTTCCGCCGTCCGCCTGGTCGCCAAG-ATGCCGACCCTGGCA

>CC1583_PG10a

CACCGGCCGCTTCACATTTGACCCTGGCTTC-ATGTCCACGGCATC-TTGCGAGTCGAAGATCACCTACATCGATGGTGATAACGGAATCCTGCTTCACCGCGGCTACCCGATCGAGCAACTGGCCGAGCAGTCCGACTATCTGGAAA-CCTGCTACCTGCTGCTCAATGGCGAACTGCCAACTGCCGAGCAGAAAGCCCAGTTCGTTGCCGTGGTCAAGAACCACACGATGGTTCACGAACAGCTCAAGACCTTTTTCAACGGCTTCCGCCGTGATGCCCACCCGATGGCCGTCATGTGCGGCGTGGTCGGGGCCCTGTCGGCGTTCTATCACGACTCGCTGGACATCAATAACCCGCAGCACCGCGAGATTTCTGCCGTGCGCCTGGTCGCCAAG-ATGCCGACCCTGGCA

>CCE0103_PG10a

CACCGGCCGCTTCACATTTGACCCTGGCTTC-ATGTCCACGGCATC-TTGCGAGTCGAAGATCACCTACATCGATGGTGATAACGGAATCCTGCTTCACCGCGGCTACCCGATTGAGCAACTGGCCGAGCAGTCCGATTATCTGGAAA-CCTGCTACCTGCTGCTCAATGGCGAACTGCCAACTGCCGAGCAGAAAGCCCAGTTCGTTGCCGTGGTCAAGAACCACACGATGGTTCACGAACAGCTCAAGACCTTTTTCAACGGCTTCCGCCGTGATGCCCACCCGATGGCCGTCATGTGCGGCGTGGTCGGTGCCCTGTCGGCGTTCTATCACGACTCGCTGGACATCAATAACCCGCAGCACCGCGAGATTTCTGCCGTGCGTCTGGTCGCCAAG-ATGCCGACCCTGGCA

>USA0102_PG10a

CACCGGCCGCTTCACATTTGACCCTGGCTTC-ATGTCCACGGCATC-TTGCGAGTCGAAGATCACCTACATCGATGGTGATAACGGAATCCTGCTTCACCGCGGCTACCCGATCGAGCAACTGGCCGAGCAGTCCGATTATCTGGAAA-CCTGCTACCTGCTGCTCAATGGCGAACTGCCAACTGCCGAGCAGAAAGCCCAGTTCGTTGCCGTGGTCAAGAACCACACGATGGTTCACGAACAGCTCAAGACCTTTTTCAACGGCTTCCGCCGTGATGCCCACCCGATGGCCGTCATGTGCGGCGTGGTCGGTGCCCTGTCGGCGTTCTATCACGACTCGCTGGACATCAATAACCCGCAGCACCGCGAGATTTCTGCCGTGCGCCTGGTCGCCAAG-ATGCCGACCCTGGCA

>TA0019_PG10b

CACCGGCCGCTTCACATTTGACCCTGGCTTC-ATGTCCACGGCATC-TTGCGAGTCGAAGATCACCTACATCGATGGTGATAACGGAATTCTGCTTCACCGCGGCTACCCGATCGAACAACTGGCCGAGCAGTCCGATTATCTGGAAA-CCTGCTACCTGCTGCTCAATGGCGAATTGCCAACCGCCGAGCAGAAAGCCCAGTTCGTTGCCGTGGTCAAAAACCACACCATGGTTCACGAACAGCTCAAGACCTTTTTCAACGGTTTCCGCCGTGATGCCCACCCGATGGCCGTCATGTGCGGCGTAGTCGGCGCCCTGTCGGCGTTCTATCACGACTCGCTGGACATCAACAACCCGCAACACCGCGAGATTTCTGCAGTGCGCCTGGTCGCCAAG-ATGCCGACCCTGGCA

>CLA0275_PG10b

CACCGGCCGCTTCACATTTGACCCTGGCTTC-ATGTCCACGGCATC-TTGCGAGTCGAAGATCACCTACATCGATGGTGATAATGGAATTCTGCTTCACCGCGGCTACCCGATCGAACAACTGGCCGAGCAGTCCGATTATCTGGAAA-CCTGCTACCTGCTGCTTAATGGCGAATTGCCAACCGCCGAGCAGAAAGCCCAGTTCGTTGCCGTGGTCAAAAACCACACGATGGTTCACGAACAGCTCAAGACCTTTTTCAACGGTTTCCGCCGTGATGCCCACCCGATGGCCGTCATGTGCGGCGTAGTCGGCGCCCTGTCGGCGTTCTATCACGACTCGCTGGACATCAATAACCCGCAGCACCGCGAGATTTCTGCCGTGCGCCTGGTCGCCAAG-ATGCCGACCCTGGCA

>TA0003_PG10b

CACCGGCCGCTTCACATTTGACCCTGGCTTC-ATGTCCACGGCATC-TTGCGAGTCGAAGATCACCTACATCGATGGTGATAACGGAATTCTGCTTCACCGCGGCTACCCGATCGAACAACTGGCCGAGCAGTCCGACTATCTGGAAA-CCTGCTACCTGCTGCTCAATGGCGAATTGCCAACCGCCGAGCAGAAAGCCCAGTTCGTTGCCGTGGTCAAAAACCACACGATGGTTCACGAACAGCTCAAGACCTTTTTCAACGGTTTCCGCCGTGATGCCCACCCGATGGCCGTCATGTGCGGCGTAGTCGGCGCCCTGTCGGCGTTCTATCACGACTCTCTGGACATCAACAACCCGCAGCACCGCGAGATTTCTGCCGTGCGCCTGGTCGCCAAG-ATGCCGACCCTGGCA

>CC1586_PG10c

CACCGGCCGCTTCACATTTGACCCTGGCTTC-ATGTCCACGGCATC-TTGCGAGTCGAAGATCACCTACATCGATGGTGATAACGGAATCCTGCTTCACCGCGGCTACCCGATCGAACAACTGGCCGAGCAGTCCGATTATCTGGAAA-CCTGCTACCTGCTGCTCAATGGCGAATTGCCGACCGCGGAGCAAAAAGCCCAGTTCGTTGCCGTGGTCAAAAACCACACAATGGTTCACGAACAGCTCAAGACCTTTTTCAACGGCTTCCGCCGTGATGCCCACCCGATGGCCGTCATGTGCGGCGTAGTCGGCGCCCTGTCGGCGTTCTATCACGACTCGCTGGACATCAACAACCCGCAGCACCGCGAGATTTCTGCCGTGCGCCTGGTCGCCAAG-ATGCCGACCCTGGCA

>CCE0100_PG10d

CACCGGCCGCTTCACATTTGACCCTGGCTTC-ATGTCCACGGCATC-TTGCGAGTCGAAGATCACCTACATCGATGGTGATAACGGAATCCTGCTTCACCGCGGCTACCCGATCGAACAACTGGCCGAGCAGTCCGATTATCTGGAAA-CCTGCTACCTGCTGCTAAATGGCGAATTGCCAACAGCCGAGCAAAAAGCCCAGTTCGTTGCCGTGGTCAAGAACCACACGATGGTTCACGAACAGCTCAAGACCTTTTTCAACGGCTTCCGCCGTGATGCCCACCCGATGGCCGTCATGTGCGGCGTAGTCGGCGCCCTGTCGGCGTTCTATCACGACTCGCTGGACATCAATAACCCCCAGCACCGCGAGATTTCTGCCGTGCGCCTGGTCGCCAAG-ATGCCGACCCTGGCA

>USA0032_PG10e

CACCGGCCGCTTCACATTTGACCCTGGCTTC-ATGTCCACGGCATC-TTGCGAGTCGAAGATCACCTACATCGACGGTGATAACGGAATCCTGCTTCACCGCGGCTACCCGATCGAACAACTGGCCGAGCAGTCCGATTACCTGGAAA-CCTGCTACCTGTTGCTCAATGGCGAATTGCCAACTGCCGAGCAGAAAGCCCAGTTCGTTGCCGTGGTCAAGAACCACACGATGGTTCACGAACAGCTCAAGACCTTTTTCAACGGCTTCCGCCGTGATGCCCACCCGATGGCCGTCATGTGCGGCGTAGTCGGCGCGCTGTCGGCGTTCTATCACGACTCGCTGGACATCAACAACCCGCAGCACCGCGAGATTTCTGCCGTGCGCCTGGTCGCCAAG-ATGCCGACCCTGGCA

>CCE0153_PG10f

CACCGGCCGCTTCACATTTGACCCTGGCTTC-ATGTCCACGGCATC-TTGCGAGTCGAAGATCACCTACATCGATGGTGATAACGGAATCCTGCTTCACCGCGGCTACCCGATCGAACAACTGGCCGAGCAGTCCGATTATCTGGAAA-CCTGCTACCTGCTGCTCAATGGCGAATTGCCAACTGCCGAGCAGAAAGCCCAGTTCGTTGCCGTGGTCAAGAACCACACGATGGTTCACGAACAGCTCAAGACCTTTTTCAACGGCTTCCGCCGTGATGCCCACCCGATGGCCGTCATGTGCGGCGTAGTCGGCGCCCTGTCGGCGTTCTATCACGACTCGCTGGACATCAATAACCCGCAGCACCGCGAGATTTCTGCCGTGCGCCTGGTCGCCAAG-ATGCCGACCCTGGCA

>CCV0213_PG10g

CACCGGCCGCTTCACATTTGACCCTGGCTTC-ATGTCCACGGCATC-TTGCGAGTCGAAGATCACCTACATTGATGGTGATAACGGAATCCTGCTTCACCGCGGCTACCCGATCGAGCAACTGGCCGAGCAGTCCGATTATCTGGAAA-CCTGCTACCTGCTGCTCAACGGCGAACTGCCGACTGCCGAGCAGAAAGCGCAGTTCGTTGCTGTGGTCAAGAACCACACGATGGTTCACGAACAGCTCAAGACCTTTTTCAACGGCTTCCGCCGTGATGCCCACCCGATGGCCGTCATGTGCGGCGTGGTCGGCGCCCTGTCGGCGTTCTATCACGACTCGCTGGACATCAATAACCCGCAGCACCGCGAGATTTCTGCCGTGCGCCTGGTTGCCAAG-ATGCCGACCCTGGCA

>83.1_PG11

CACCGGCCGCTTCACATTCGACCCTGGCTTC-ATGTCGACCGCCTC-TTGCGAGTCGAAGATCACCTACATTGATGGTGACAATGGCATTCTGCTGCACCGCGGTTATCCGATCGAGCAACTGGCCGAACAGTCCGATTATCTGGAAA-CCTGCTACCTTCTGCTCAACGGCGAATTGCCGACCAGCGAACAGAAAGCCCAGTTCGTTGCCGTGGTCAAGAACCACACCATGGTTCACGAACAGCTGAAGAGCTTCTTCAACGGCTTCCGCCGCGACGCCCACCCGATGGCTGTCATGTGCGGCGTGGTTGGTGCCCTGTCCGCGTTCTACCACGACTCGCTGGACATCAATAACCCGCAACACCGCGAAATTTCTGCAGTGCGCCTGGTCGCCAAG-ATGCCGACCCTGGCA

>CFBP4407_PG11

CACCGGCCGCTTCACATTCGACCCTGGCTTC-ATGTCGACCGCCTC-TTGCGAGTCGAAGATCACCTACATTGATGGTGACAATGGCATTCTGCTGCACCGCGGTTATCCGATCGAGCAACTGGCCGAACAGTCCGATTATCTGGAAA-CCTGCTACCTTCTGCTCAATGGCGAACTGCCAACCAGCGAACAGAAAGCCCAGTTCGTTGCTGTGGTCAAGAACCACACCATGGTCCACGAGCAGCTCAAGAGCTTCTTCAACGGATTCCGTCGCGACGCCCACCCGATGGCCGTCATGTGTGGCGTAGTCGGTGCACTGTCCGCGTTTTACCACGACTCGCTGGACATCAATAACCCGCAACACCGTGAAATCTCGGCAGTTCGACTGGTCGCCAAG-ATGCCGACCCTGGCA

>GAW0112_PG12a

CACCGGCCGCTTCACATTCGACCCAGGCTTC-ATGTCGACCGCGTC-TTGCGAGTCGAAGATCACCTACATTGACGGTGACAACGGGATCCTGCTGCATCGCGGCTATCCGATCGAGCAATTGGCCGAGCAGTCGGACTATCTCGAAA-CCTGCTACCTGCTGCTCAACGGTGAACTGCCAACCGCTGAACAGAAAGCCCAGTTCGTGGTTGTGGTCAAGAACCACACAATGGTTCACGAACAGCTGAAGACTTTCTTCAACGGCTTCCGCCGTGACGCTCACCCGATGGCGGTGATGTGCGGCGTAGTCGGAGCCTTGTCGGCGTTTTATCACGACTCCCTGGATATCAATAATCCTCAGCATCGTGAAATCTCGGCCATTCGCCTCGTGGCGAAG-ATGCCGACCCTGGCA

>GAW0113_PG12b

CACCGGCCGCTTCACATTCGACCCAGGCTTC-ATGTCGACCGCGTC-TTGCGAGTCGAAGATCACCTACATTGACGGTGACAACGGGATCCTGCTGCATCGCGGCTATCCGATCGAGCAACTGGCCGAACAGTCGGACTATCTCGAAA-CCTGCTACCTGCTGCTCAACGGTGAACTGCCAACCGCTGAACAGAAAGCCCAGTTCGTGGTTGTGGTCAAGAACCACACGATGGTCCACGAACAGCTGAAGACTTTCTTCAACGGCTTCCGTCGTGACGCTCACCCAATGGCGGTGATGTGCGGCGTGGTCGGAGCGCTGTCGGCGTTCTATCACGACTCTCTGGATATCAATAATCCCCAGCATCGTGAAATCTCGGCCATTCGCCTCGTGGCGAAG-ATGCCAACCCTGGCA

>CCE0915_PG13a

CACCGGCCGCTTCACATTCGACCCAGGCTTC-ATGTCGACCGCCTC-TTGCGAGTCGAAGATCACCTACATTGATGGTGACAACGGGATCCTGCTGCACCGCGGCTATCCGATCGAACAACTGGCCGAGCAATCGGACTATCTGGAAA-CCTGCTATCTGCTGCTAAACGGCGAACTGCCAACCGCCGAGCAGAAAGCCCAGTTCGTTGTCGTCGTGAAGAACCACACCATGGTTCACGAACAGCTCAAGACCTTCTTCAACGGCTTCCGCCGCGACGCTCACCCGATGGCGGTGATGTGTGGGGTGGTGGGTGCCCTCTCGGCGTTCTATCACGACTCTCTGGACATCAATAATCCACAGCACCGTGAAATCTCGGCCATCCGTCTGGTCGCCAAG-ATGCCGACGCTGGCA

>UB246_PG13a

CACCGGCCGCTTCACATTCGACCCAGGCTTC-ATGTCGACCGCCTC-TTGCGAGTCGAAGATCACCTACATTGATGGTGACAACGGGATCCTGCTGCACCGCGGCTATCCGATCGAACAACTGGCCGAGCAATCGGACTACCTGGAAA-CGTGCTATCTGCTACTGAACGGCGAGCTGCCAACCGCCGAGCAGAAAGCCCAGTTCGTTGTTGTCGTGAAAAACCACACCATGGTTCACGAACAGCTCAAGACCTTTTTCAACGGCTTCCGCCGCGACGCTCACCCGATGGCGGTGATGTGTGGTGTGGTAGGTGCCCTCTCGGCGTTCTATCACGACTCTCTGGACATCAATAATCCACAGCACCGTGAAATCTCGGCCATCCGTCTGGTCGCCAAG-ATGCCGACGCTGGCA

>CLA0302_PG13b

CACAGGCCGCTTCACATTCGACCCAGGCTTC-ATGTCGACCGCCTC-TTGCGAGTCGAAGATCACCTACATTGATGGTGACAACGGGATCCTGCTGCACCGCGGCTACCCGATCGAACAATTGGCCGAGCAGTCGGACTACCTGGAAA-CCTGCTATCTGCTGCTGAACGGCGAACTGCCAACCGCTGAGCAGAAAGCCCAGTTCGTTGTTGTCGTCAAGAACCACACCATGGTTCACGAACAGCTCAAGACCTTTTTCAACGGCTTCCGCCGCGACGCTCACCCGATGGCGGTGATGTGTGGTGTGGTGGGTGCCCTTTCCGCGTTCTATCACGACTCTCTGGACATCAATAATCCACAGCATCGTGAAATCTCGGCCATTCGTCTGGTCGCCAAG-ATGCCGACCCTGGCA

>CCV0567_PG13b

CACCGGCCGCTTCACATTCGACCCAGGCTTT-ATGTCGACCGCCTC-TTGCGAGTCGAAGATCACCTACATTGATGGTGACAACGGGATTCTGCTGCACCGCGGCTATCCGATCGAACAATTGGCCGAGCAATCGGACTACCTGGAAA-CCTGCTATCTGCTGCTGAACGGCGAACTGCCAACCGCCGAGCAGAAAGCCCAGTTCGTGGTTGTCGTAAAGAACCACACCATGGTTCACGAACAACTCAAGACCTTTTTCAACGGCTTCCGCCGCGACGCTCACCCGATGGCGGTGATGTGTGGTGTGGTGGGTGCCCTTTCCGCGTTCTATCACGACTCTCTGGACATCAATAATCCACAGCATCGTGAAATCTCGGCCATTCGTCTGGTCGCCAAG-ATGCCGACGCTGGCA

>38B9_P.graminis

CACCGGACGCTTCACATTCGACCCAGGCTTC-ATGTCCACCGCTTC-ATGCGAATCGAAGATCACCTACATCGATGGTGACAACGGCATTCTCCTGCATCGCGGCTACCCGATCGAACAGCTGGCCGAGCAATCGGACTACCTGGAAA-CCTGCTACCTGCTGCTCAACGGCGAACTGCCGACCGCCGAGCAAAAGGCCGAATTCGTCGTCACCGTGAAGAATCACACCATGGTGCACGAGCAACTCAAGACCTTCTTCAACGGTTTCCGTCGCGACGCCCACCCGATGGCCGTCATGTGTGGCGTGGTCGGTGCCCTCTCGGCGTTCTACCACGACTCCCTGGACATCAATAACCCGCAGCACCGCGAAATCTCCGCGATCCGCCTGGTCGCCAAG-ATGCCGACCCTGGCC

>6B4_P.rhizosphaerae

CACCGGACGCTTCACATTCGACCCAGGCTTC-ATGTCCACCGCCTC-ATGCGAATCCAAGATCACCTACATCGACGGTGACAACGGCATCCTGCTGCACCGCGGCTACCCGATCGAGCAACTGGCCGAGCAGTCGGACTACCTCGAAA-CCTGCTACCTGCTGCTCAACGGCGAATTGCCGACTGCCGAGCAGAAGGCGCAATTCGTCGTCACCGTGAAGAATCACACCATGGTGCACGAGCAGCTGAAGACCTTCTTCAACGGTTTCCGCCGCGACGCCCACCCGATGGCCGTGATGTGCGGCGTCGTCGGGGCCCTCTCGGCGTTCTACCACGACTCGCTGGACATCAATAACCCGCAACACCGCGAGATTTCCGCGATCCGCCTGGTCGCCAAG-ATGCCGACCCTGGCT

>Pf-05_P.protegens

CACGGGCCGCTTCACTTTTGACCCGGGTTTC-ATGTCGACCGCCTC-CTGCGAGTCGAAAATCACCTACATCGACGGCGACAACGGCATTCTGCTGCATCGCGGCTACCCGATCGAACAGCTGGCCGAGAAATCCGACTACCTGGAAA-CCTGCTACCTGCTGCTCAACGGTGAATTGCCCACCGCCGAACAGAAGGCCCAGTTCGTCAGCACCGTGAAAAACCACACCATGGTTCACGAGCAGTTGAAGAGCTTCTTCAACGGTTTCCGCCGCGACGCCCACCCAATGGCGGTGATGTGCGGCGTAGTGGGTGCACTCTCGGCGTTCTACCACGACTCCCTGGACATCAATAACCCACAGCATCGCGAAATCTCCGCTGTGCGCCTGGTGGCCAAG-ATGCCGACCCTGGCA

>PAO1_P.aeruginosa

CACGGGCCACTTCACCTTCGATCCTGGCTTC-ATGTCGACCGCCTC-CTGCGAGTCGAAGATCACCTATATCGACGGCGACAAAGGCGTCCTCCTCCATCGCGGCTACCCCATCGAGCAACTGGCAGAGAAATCCGACTACCTGGAAA-CCTGCTACCTGCTGCTGAACGGCGAGCTGCCCACCGCCGCGCAGAAGGAACAGTTCGTCGGCACCATCAAGAACCACACCATGGTTCACGAGCAGTTGAAGACCTTCTTCAACGGCTTCCGCCGCGACGCCCACCCGATGGCCGTGATGTGCGGCGTGATCGGCGCCCTCTCGGCCTTCTACCACGACTCCCTGGACATCAATAACCCGAAGCATCGCGAAGTCTCCGCGCATCGCCTGATCGCCAAG-ATGCCGACCATCGCC
