# Supplementary material for: A User's Guide to a Data Base of the Diversity of Pseudomonas syringae and Its Application to Classifying Strains in This Phylogenetic Complex
Source: PLoS One. 2014 Sep 3;9(9):e105547. doi: 10.1371/journal.pone.0105547 (PMC4153583; doi:10.1371/journal.pone.0105547)
Supplement: Text S1 — Supplementary Information. Selection of P. syringae strains. Validation of the cts gene as a P. syringae tool classification. Characterization of ice nucleation activity. Characterization of syringomycin-like toxin production. Characterization of pathogenicity and aggressiveness of P. syringae strains. (DOCX) [file pone.0105547.s015.docx]

**Text S1 : Supplementary information**

**Selection of *P. syringae* strains**

Strains collected here do not represent endangered species. Strains collected from cultivated plants were collected in the course of diagnostics of samples provided by farmers or by field technicians in the context of collaborative research. All strains included in the lab collection were purified before being stored at – 80° C in 40 % glycerol and they all lacked arginine dihydrolase and cytochrome *c* oxidase activity except for the *P. cichorii* reference strains that are oxidase positive. Strains varied in their production of fluorescent pigment on King’s medium B (KB) and in induction of a hypersensitive reaction (HR) on tobacco.

Over the past several years the housekeeping gene encoding for citrate synthase, *cts* (also named *glt*A) was sequenced for a subset of 1630 strains (among the 7000 strains from the collection) isolated from environmental habitats and crops [1, 2]. During the exploratory work of our studies, construction of a phylogenetic tree based on the 1630 *cts* sequences allowed us to select strains that represented the range of genetic diversity within each of the phylogroups and clades that could be delimited in the first-approximation analysis (unpublished data).

This led us to select the 763 strains isolated from fresh water and epilithic biofilms (56 %), snowpack (16 %), plants (11 %), precipitation (9 %), and litter (8 %) that were subsequently characterized for 12 phenotypic traits typically used in characterization of *P. syringae* (see Table S1) thereby allowing us to evaluate phenotypic diversity within different genetic groups. We selected many non-plant derived strains to better describe the unknown phylogenetic groups of the *P. syringae* complex.

The total of 836 strains used in this study is listed in Table S1. Among them 6 were reference strains chosen outside of the *P. syringae* group of strains mainly for phylogenetic analyses. The 830 remaining strains of *P. syringae* consisted of the 763 phenotyped strains and 67 strains from public data-bases used for their MLST profiles and not phenotyped here. From the 763 characterized strains, 149 were selected to cover the maximum variability observed for MLST. To this data-base of 149 MLST-typed (4 genes) strains we added the MLST profiles of the 67 strains from public data bases. The pooled set of 216 MLST profiles was used to construct more robust trees and to evaluate the reliability of phylogenetic predictions based on single housekeeping genes vs. combined gene sequences.

The affiliation to phylogroups of the remaining 614 strains of *P. syringae* was based on *cts* sequence analysis. Finally, a set of 29 strains (Table S1) that represented the maximum diversity among the *P. syringae* genomes available in GenBank and chosen from the set of 216 MLST strains, was used to compare phylogenetic positioning of strains based on core genome sequences vs. that based on single and multiple housekeeping genes.

**Validation of the *cts* gene as *P. syringae* tool classification**

To validate the choice of the *cts* gene, we first compared the phylogenetic affiliation of the set of 216 MLST-typed strains (see “Results and discussion”) using each of the four single housekeeping genes sequences as follow: i) for each housekeeping gene, a matrix of genetic distances was built (see Tables S2, S3, S4 and S5). Criteria were established for finding the phylogroups and clades already delineated in the MLST analysis (see Table S7); For example the minimum distance between strains within *cts*-phylogroups is limited to 4 % (previously 5 % within MLST-phylogroups) and 1.8 % within cts-clades (previously 2.3 % within MLST-clades) (Table S6); and ii) For each of the four matrixes of distances (Tables S2, S3, S4 and S5), the 216 strains were individually affiliated to one phylogroup and clade by looking at the minimum distance of each strain with the remaining 215 strains. Strains were classified in the same group than the closest strain in the limit of the thresholds established in i). As shown on Table S6, the *cts* and *gapA* analyses show the least difference with the affiliation obtained in the MLST analysis, (respectively 3 and 2 strains wrongly classified) compared to the analyses with *gyrB* and *rpoD* genes (respectively 12 and 9 strains misclassified). We selected the *cts* sequence to be used to classified strains with one single gene because it was largely used in previous studies [1]. The classification of the 216 strains were confirmed on the phylogenetic tree built with the single *cts* sequences (Fig S1-B).

**Characterization of ice nucleation activity (INA)**

Bacterial suspensions (10^7^ CFU ml^-1^) obtained from cultures growth on KB for three days at 26 °C were used to test INA. For each strain, three drops of 20 µl were deposited on an aluminum plate floating on a cooling bath as described previously [3]. Freezing was determined at one degree intervals from - 2 °C to - 8 °C. Freezing was scored as positive when at least two of the droplets froze.

**Characterization of syringomycin-like toxins production**

Strains were tested for production of syringomycin-like toxins based on a bioassay with *Geotricum candidum* and the SRM medium as described previously [4]. Bacteria were grown for eight days on SRM agar plate, before the plate was sprayed with a suspension of *G. candidum*, The inhibition zone around the bacterial colony was measured after two days as the distance between the edge of the fungus and the bacterial colony.

**Characterization of pathogenicity and aggressiveness**

*Cucumis melo* var. *cantalupensis* Naud. cv. Vedrantais seedlings were used as an indicator plant to estimate the pathogenicity and the level of aggressiveness. These parameters were assessed after infiltrated twelve seedlings at the junction of the cotyledons, 10 µl of an aqueous bacterial suspension (10^8^ CFU ml^-1^) prepared from 48 h bacterial cultures [2]. Seedlings were incubated for seven days with a photoperiod of 16 h of light at 21 °C during the day and 18 °C during the dark period. Symptoms on seedlings were scored as follows: 0 (no symptoms), 1 (one cotyledon with necrosis or completed wilted), 2 (necrosis on both cotyledons), 3 (both cotyledons wilted and stem symptoms) and 4 (death of the cotyledons and stem). Pathogenicity was recorded positive when the frequency of seedlings with symptoms (F) was > 50 % and aggressiveness was calculated as the mean score of symptoms (µ) (see Table S1).

**References**

[1] Morris CE, Sands DC, Vanneste JL, Montarry J, Oakley B, et al. (2010) Inferring the evolutionary history of the plant pathogen *Pseudomonas syringae* from its biogeography in headwaters of rivers in North America, Europe and New Zealand. MBio 1: 00107–00110.

[2] Morris CE, Sands DC, Vinatzer BA, Glaux C, Guilbaud C, et al. (2008) The life history of the plant pathogen *Pseudomonas syringae* is linked to the water cycle. ISME J 2: 321–334.

[3] Morris CE, Kinkel LL, Xiao K, Prior P, Sands DC (2007) Surprising niche for the plant pathogen *Pseudomonas syringae*. Infect Genet Evol 7: 84–92.

[4] Xu GW, Gross DC (1988) Evaluation of the Role of Syringomycin in Plant Pathogenesis by Using Tn5 Mutants of *Pseudomonas syringae* pv. *syringae* Defective in Syringomycin Production. Appl Environ Microbiol 54: 1345–1353.
